# Regulation of Mycolactone, the *Mycobacterium ulcerans* Toxin, Depends on Nutrient Source

**DOI:** 10.1371/journal.pntd.0002502

**Published:** 2013-11-14

**Authors:** Caroline Deshayes, Shiva Kumar Angala, Estelle Marion, Irène Brandli, Jérémie Babonneau, Laurent Preisser, Sara Eyangoh, Yves Delneste, Pierre Legras, Chantal De Chastellier, Timothy P. Stinear, Mary Jackson, Laurent Marsollier

**Affiliations:** 1 LUNAM, Université d'Angers, Angers, France; 2 ATOMycA, Inserm Avenir Team, CRCNA, Inserm U892, 6299 CNRS Angers, Angers, France; 3 Mycobacteria Research Laboratories, Department of Microbiology, Immunology and Pathology, Colorado State University, Fort Collins, Colorado, United States of America; 4 Centre d'Immunologie de Marseille-Luminy (CIML), Inserm UMR 1104, CNRS UMR 7280, Aix-Marseille University UM 2, Marseille, France; 5 Laboratoire de bactériologie, CHU Angers, Angers, France; 6 Team 7A, CRCNA Inserm U892, 6299 CNRS Angers, Angers, France; 7 Laboratoire des Mycobactéries et Jeune Equipe associée IRD ATOMyC, Centre Pasteur du Cameroun, Yaoundé, Cameroun; 8 CHU Angers, Laboratoire d'immunologie, Angers, France; 9 SCAHU, Faculté de Médecine et CHU, Angers, France; 10 Department of Microbiology and Immunology, University of Melbourne, Melbourne, Victoria, Australia; Kwame Nkrumah University of Science and Technology, Ghana

## Abstract

**Background:**

*Mycobacterium ulcerans*, a slow-growing environmental bacterium, is the etiologic agent of Buruli ulcer, a necrotic skin disease. Skin lesions are caused by mycolactone, the main virulence factor of *M. ulcerans*, with dermonecrotic (destruction of the skin and soft tissues) and immunosuppressive activities. This toxin is secreted in vesicles that enhance its biological activities. Nowadays, it is well established that the main reservoir of the bacilli is localized in the aquatic environment where the bacillus may be able to colonize different niches. Here we report that plant polysaccharides stimulate *M. ulcerans* growth and are implicated in toxin synthesis regulation.

**Methodology/Principal Findings:**

In this study, by selecting various algal components, we have identified plant-specific carbohydrates, particularly glucose polymers, capable of stimulating *M. ulcerans* growth *in vitro*. Furthermore, we underscored for the first time culture conditions under which the polyketide toxin mycolactone, the sole virulence factor of *M. ulcerans* identified to date, is down-regulated. Using a quantitative proteomic approach and analyzing transcript levels by RT-qPCR, we demonstrated that its regulation is not at the transcriptional or translational levels but must involve another type of regulation. *M. ulcerans* produces membrane vesicles, as other mycobacterial species, in which are the mycolactone is concentrated. By transmission electron microscopy, we observed that the production of vesicles is independent from the toxin production. Concomitant with this observed decrease in mycolactone production, the production of mycobacterial siderophores known as mycobactins was enhanced.

**Conclusions/Significance:**

This work is the first step in the identification of the mechanisms involved in mycolactone regulation and paves the way for the discovery of putative new drug targets in the future.

## Author Summary

Mycolactone, a polyketide cytotoxic toxin, is the key virulence factor responsible for large skin ulcers in Buruli ulcer. This disease, mainly occurring in humid tropical zones, especially in West African countries, is due to infection by *Mycobacterium ulcerans*, a slow-growing environmental mycobacterium. The toxin has destructive effects on the skin, soft tissues and bones. In this study, we brought out for the first time that addition of specific carbohydrate to culture medium induces the down-regulation of the toxin. Furthermore, this decrease in toxin production is correlated with the activation of the iron acquisition pathway, especially by siderophore production. These results show that *M. ulcerans* adapts its metabolism to culture conditions, which probably reflect its adaptation in its natural habitats. This work is the first step in toxin regulation understanding which is a key issue for a better comprehension of Buruli ulcer physiopathology and the identification of putative therapeutic targets.

## Introduction

*Mycobacterium ulcerans*, a slow-growing environmental bacterium, is the causative agent of Buruli ulcer, a severe infectious skin disease. This disease mainly occurs in humid tropical zones, especially in West African countries. Its incidence is increasing and this disease became the third mycobacteriosis after tuberculosis and leprosy has been declared as a (re-)emerging disease by the World Health Organization [1], [2].

The massive tissue destructions forming large painless ulcers with undermined edges that could touch the bone tissue are induced by a bacterial toxin, the mycolactone, which remains, the main virulence factor [3], [4]. This polyketide toxin has intense cytotoxic activity *in vitro*, affecting numerous cell types [5], is thought to have immune-modulatory activities decreasing the efficiency of the immune system [6] and inhibits *M. ulcerans* uptake by phagocytes, which led to the interpretation that *M. ulcerans* was an extracellular pathogen. However, an intra-macrophage growth phase for those bacilli has been described [7], showing that the production of a cytotoxic exotoxin can be conciliated with an intracellular lifestyle. The authors suggested that *M. ulcerans* probably turns off the mycolactone synthesis during the intra-macrophage growth. The genes encoding the six enzymes involved in the toxin synthesis are located on a giant plasmid [8], [9], [10], [11]. As various mycobacterial species [12], *M. ulcerans* produces membrane vesicles which are the main reservoir of mycolactone. Depending on its environment, these vesicles are engulfed in an extracellular matrix produced by *M. ulcerans* [13].

Based on the existence of foci of Buruli ulcer cases in people living in swampy or poorly drained areas in Africa, the aquatic environment has long been thought to be a reservoir for this mycobacterium [14], [15], [16]. However, the environmental ecology of *M. ulcerans* remained obscure for a long time due to the failure to isolate the bacteria in culture from the environment [13], [17], [18], [19], [20], [21], [22]. Nowadays, it is well established that the reservoir of *M. ulcerans* is localized in the aquatic environment where the bacillus may colonize different niches. Nevertheless, despite decades of research, the mode of transmission of *M. ulcerans* remains unclear and several hypotheses about potential reservoirs, vectors, and transmission mechanisms have been proposed [23], [24], [25], [26], [27]. A conceptual model of food web based on the role of water bugs as specific hosts and potential vectors of the bacilli has been proposed [21], [28], [29], [30], [31], [32], [33], [34], [35]. The role of human biting water bugs in *M. ulcerans* transmission was recently strengthened by the detection of viable bacilli in their saliva in an environmental study [29].

In aquatic environments bacteria are predominantly not free floating but grow as multi-species communities attached to submerged surfaces [36], [37]. This seems to be the case for *M. ulcerans* which has been shown to grow as dense clusters on the surface of aquatic plants [22]. Furthermore, crude extracts from the green algae *Rhizoclonium* sp. and *Hydrodictyon reticulatum* have been shown to halve the doubling time of *M. ulcerans in vitro*. Aquatic plants, such as algae, are able to secrete many organic compounds, including polysaccharides, which are used by bacteria as substrates for growth [38], [39], [40]. While other mycobacteria have the ability to form biofilms on algal particulate matter, the growth stimulation effect seems to be specific to *M. ulcerans* [22]. Indeed, this stimulation has not been observed with other slow-growing mycobacteria, suggesting that *M. ulcerans* uses specific components of the extract to augment its growth. Based on these observations, we aimed to define the carbohydrates involved in *M. ulcerans* growth stimulation. This bacterium is a slow-growing microorganism with a doubling time of 3.5 days [41] and the finding of specific supplements capable of enhancing its growth rate would indeed be a great benefit for the scientific community and diagnostic centers. By selecting various algal components, we identified polysaccharides able to stimulate *M. ulcerans* growth *in vitro*. We describe for the first time a condition under which mycolactone, *M. ulcerans*' toxin, is down-regulated. Under this specific growth condition, the production of the mycobacterial siderophore, mycobactin appears to be activated.

## Materials and Methods

### Ethics statement

#### (i) Animals experiments

All animal experiments were performed in full compliance with national guidelines (articles R214- 87 to 90 from French “code rural”) and European guidelines (Directive 2010/63/EU of the European Parliament and of the council of 22 September 2010 on the protection of animals used for scientific purposes). All protocols were approved by the ethics Committee of region Pays de la Loire under protocol number CEEA 2009.14 and CEEA 2012.145. Animals were maintained under specific pathogen free conditions in the animal house facility of the Centre Hospitalier Universitaire, Angers, France (Agreement A 49 007 002).

#### (ii) Human samples

All individuals/patients were informed about the methodology and signed an informed consent according to ethical standards. The project was also approved by the Ministry of Health in Benin (Nos Réf.: 040/05/MSP/DC/SGM/DNSP/PNLUB), by the Ethics in Research Committee of the Centre Hospitalier Universitaire d'Angers (Nos Réf.: 2005 – 67) and by the Ethics in Research Committee of the Pasteur Institute (Nos Réf.: XN/LT/CT/2005.003). Patients were de-identified and study data was analyzed anonymously.

### *In vitro* growth conditions of *M. ulcerans* 1615 strain

The *M. ulcerans* 1615 strain (Trudeau Collection Strain) originally isolated from human skin biopsies from Malaysia [3] was used to study growth stimulation by various carbohydrates. Carbohydrates (0.33%) were added to the BACTEC vials containing Middlebrook 7H12B medium as growth was monitored after inoculation of 2.10^4^ bacilli as previously described [22]. For culture on solid medium, *M. ulcerans* 1615 strain was cultivated onto 7H10 solid medium supplemented with 10% OADC (oleic acid, dextrose, catalase; Difco, Becton-Dickinson) at 30°C for one month. When required, carbohydrates were added to the medium at the appropriate concentration.

### Clinical bacterial strains and growth conditions in microMGIT

The *M. ulcerans* strains used to check the growth stimulation by starch were clinical strains isolated from Buruli patients from different endemic regions (Table 1). Among these strains, the *M. ulcerans* 1G897 strain was originally isolated from human skin biopsy from French Guiana [42]. Frozen aliquots of each strain were first inoculated onto Lowenstein-Jensen solid medium (BD). Then, 30 days later, exponentially growing bacteria from agar plates were suspended in PBS and quantified by quantitative PCR using IS*2404* primers as previously described [29]. Tubes containing BBL MGIT medium (BD BACTEC) supplemented with 7.5% of starch were completed with 0.8 ml of MGIT Growth Supplement/MGIT PANTA antibiotic mixture. Media were inoculated with 10^2^ bacteria and the fluorescence reflecting the mycobacterial growth was regularly read with the BD MicroMGIT fluorescence reader.

**Table 1:** Growth stimulation of various *M. ulcerans* strains by starch.

Strains	Geographical zone	Postive time (days)	
		MGIT	MGIT 7,5% starch
1G897	French Guiana	43.67±4.04	30.33±5.13
38	Ivory Coast	69.67±1.15	40.33±2.31
66	Ivory Coast	68.67±5.51	38.67±2.52
72	Ivory Coast	55.00±2.00	40.33±1.15
94	Ghana	77.67±6.03	50.00±0.00
128	Ghana	61.00±6.93	46.00±0.00

Clinical *M. ulcerans* isolates were grown in MGIT tubes in presence or not of starch. Fluorescence was measured over 90 days and the delay (in days) for cultures to become positive was monitored. A significant reduction of the positivity delay was observed for all strains (*p*-values<0.05 in a Student's *t*-test, except for the strain 128 from Ghana with a *p*-value of 0.064).

### Bacterial cell surface characterization of *M. ulcerans*

To quantify aggregation, a method relying on the measure of the optical density changes that occur in a non-shaking culture upon sedimentation of multicellular aggregates was used as previously described [43]. Briefly, bacteria were resuspended in PBS from agar plates and vortexed for two minutes to break the large aggregates. Unicellular mycobacteria were separated from aggregates by sedimentation (1 *g*) and the optical density at 600 nm (OD_600_) of the supernatant was measured (Genesys20, ThermoScientific) over 15 minutes and compared to the OD_600_ of resuspended cultures where the aggregates were broken up by vortexing with glass beads. The aggregative index was calculated as the ratio between the two OD_600_. Values are the mean of at least three independent experiments. For evaluation of the hydrophobic–hydrophilic character of the of *M. ulcerans* cell surface, adhesion to hexadecane, an apolar solvent, was employed as previously described [44]. The global electrical properties of the bacterial surfaces were assessed by measuring the electrophoretic mobility (EM) of bacteria that corresponds to the velocity of suspended cells under the influence of an applied electrical field [44]. Briefly, *M. ulcerans* strains was scraped from agar plates and resuspended in deionized water. The suspension was then diluted in different NaCl solutions with concentration ranging from 0 to 150 mM at constant pH = 7. Electrophoretic mobility measurements of *M. ulcerans* were carried out using a Zeta meter (Zetasizer Nano ZS, Malvern Instruments S.A., Worcestershire, UK). All EM measurements were carried out 10 times at 25°C for each strain.

### Transmission electron microscopy (TEM)

Bacteria cultivated on 7H10 medium were scraped off the plates and resuspended in PBS. They were harvested by centrifugation (8,000 *g*, 10 min), and washed twice with PBS. The resulting pellets were fixed and processed for TEM as previously described [45]. To stabilize and better visualize lipids, 0.1% Malachite green was added to some of the samples during the aldehyde and osmium fixation steps [46].

### Lipids extraction and analysis

26-d-old *M. ulcerans* cultures (75 cm^2^) were scraped from 7H10 plates supplemented with 7.5% carbohydrates and resuspended in PBS. Bacterial suspensions were centrifuged (12,000 *g*, 30 min at 4°C) and total lipids were extracted once with 2∶1 CHCl_3_/CH_3_OH and twice with 1∶2 CHCl_3_/CH_3_OH by incubation overnight at RT with stirring. After incubation, insoluble material was separated for arabinogalactan (AG) and lipoarabinomannan (LAM) preparation by spinning (3,500 rpm, 10 min), and the CHCl_3_/CH_3_OH was recovered, dried and further subjected to Folch wash. The Folch extracted total lipids were evaporated to dryness and dissolved in 200 µL of CHCl_3_. The total lipids were analyzed by TLC using aluminum-backed, 250-µm silica gel F254 plates developed in five different solvent systems, 65∶25∶4 (CHCl_3_/CH_3_OH/H_2_O), 20∶4∶0.5 (CHCl_3_/CH_3_OH/H_2_O), 60∶30∶6 (CHCl_3_/CH_3_OH/H_2_O), 98∶2 (CHCl_3_/CH_3_OH) and 98∶2 (petroleum ether/ethyl acetate). After chromatography, TLCs were sprayed separately with CuSO_4_ followed by heating.

Mycolactone quantification was performed from total lipids sample analysis by High Performance Liquid Chromatography (HPLC) as previously described [47]. The quantity of mycolactone was reported to the quantity of proteins extracted in the corresponding sample. One-way ANOVA analysis of variance was used to compare mean values between groups followed by Newman-Keuls multiple comparison test to detect significant mean differences between pairs of groups.

### Proteomic analysis by NanoLC MS/MS

26-d-old *M. ulcerans* cultures (75 cm^2^) were scraped from plate and resuspended in PBS. Bacterial suspensions were centrifuged (12,000 *g*, 30 min at 4°C). The mycobacterial pellets were then resuspended in lysis buffer (8 M urea 100 mM HEPES, pH 8.0). Following 20 sec sonication, bacteria were broken with 100-µm glass beads (Sigma) two times for 1 min at speed 30 using a bead-beater (MM200, Retsch) at 4°C. Cell debris were removed by centrifugation at 8,000 *g* at 4°C and the supernatant were kept at −80°C. Proteins quantification was performed by using Micro BCA™ Protein Assay kit (Thermo Scientific Pierce). Triplicate protein samples (400 mg) were analyzed by NanoLC MS/MS analysis using an on-line system consisting of a micro-pump Agilent 1200 binary HPLC system (Agilent Technologies, Palo Alto, CA) coupled to an LTQ-Orbitrap XL hybrid mass spectrometer (ThermoFisher, San Jose, CA) (see Material and Methods S1).

### Transcriptional analysis by RT-QPCR

*M. ulcerans* cultures were scraped from plate and resuspended in PBS. An aliquot of 1 ml was mixed to 2 ml of RNAprotect Bacteria Reagent (Qiagen) and incubated for 5 min at RT. After centrifugation (12,000 *g*, 10 min at 4°C), pellets were stored at −80°C until use. Frozen pellets were resuspended in RLT Buffer (Qiagen) containing 1% β-mercaptoethanol and bacteria were broken with 100-µm glass beads (Sigma) two times for 1 min at speed 30 using a bead-beater (MM200, Retsch). After centrifugation at 12,000 *g* for 10 min at 4°C, the supernatants were collected and RNA were purified with a RNeasy Mini Kit (Qiagen) and eluted in RNase- and DNase-free water. RNA were treated with 10 U RQ1 RNase-Free DNase (Promega) for 30 min at 37°C, repurified using RNeasy Mini Kit, eluted in 35 µl of water. The first-strand cDNA was synthesized in 20 µl reaction volumes using 500 ng of total RNA, 500 ng of random primers (Invitrogen) and the SuperScript II Reverse Transcriptase (Invitrogen). Quantitative real-time PCR was performed in 10 µl reaction volumes with iQ SYBR Green Supermix (Bio-rad), 500 nM of primers (Table S1) and 5 µl of 10 times diluted cDNA. Reactions were run on a PTC 200 thermocycler (Bio-rad) using the following program: 3 min at 95°C and 40 cycles of 10 s at 95°C and 1 min at 57°C. Analysis was performed utilizing the ΔΔCt method with the *ppk* gene as housekeeping gene as already used [48], [49].

### Virulence assays in mice

Bacteria grown on different carbohydrates were quantified by quantitative PCR using IS*2404* primers as previously described [29]. Suspensions (30 µl) containing 1.10^3^ bacteria were injected subcutaneously into the tail of 6-week-old female Balb/c mice (Janvier, Breeding Company, Le Genest, France). Mice tails were examined weekly over five months. For each condition, 7 mice were infected.

### Purification and identification of the orange pigment

To purify the orange pigment, approximately 2 mg of Folch extracted total lipids was subjected to preparative TLC using (CHCl_3_/CH_3_OH; 98∶2) as the solvent system. The chloroform extracted material subjected to LC/MS analysis in the positive mode using the method developed by Sartain *et al.* [50].

## Results

### *M. ulcerans* growth is stimulated by glucose polymers, main components of aquatic plants

**Figure 1:**
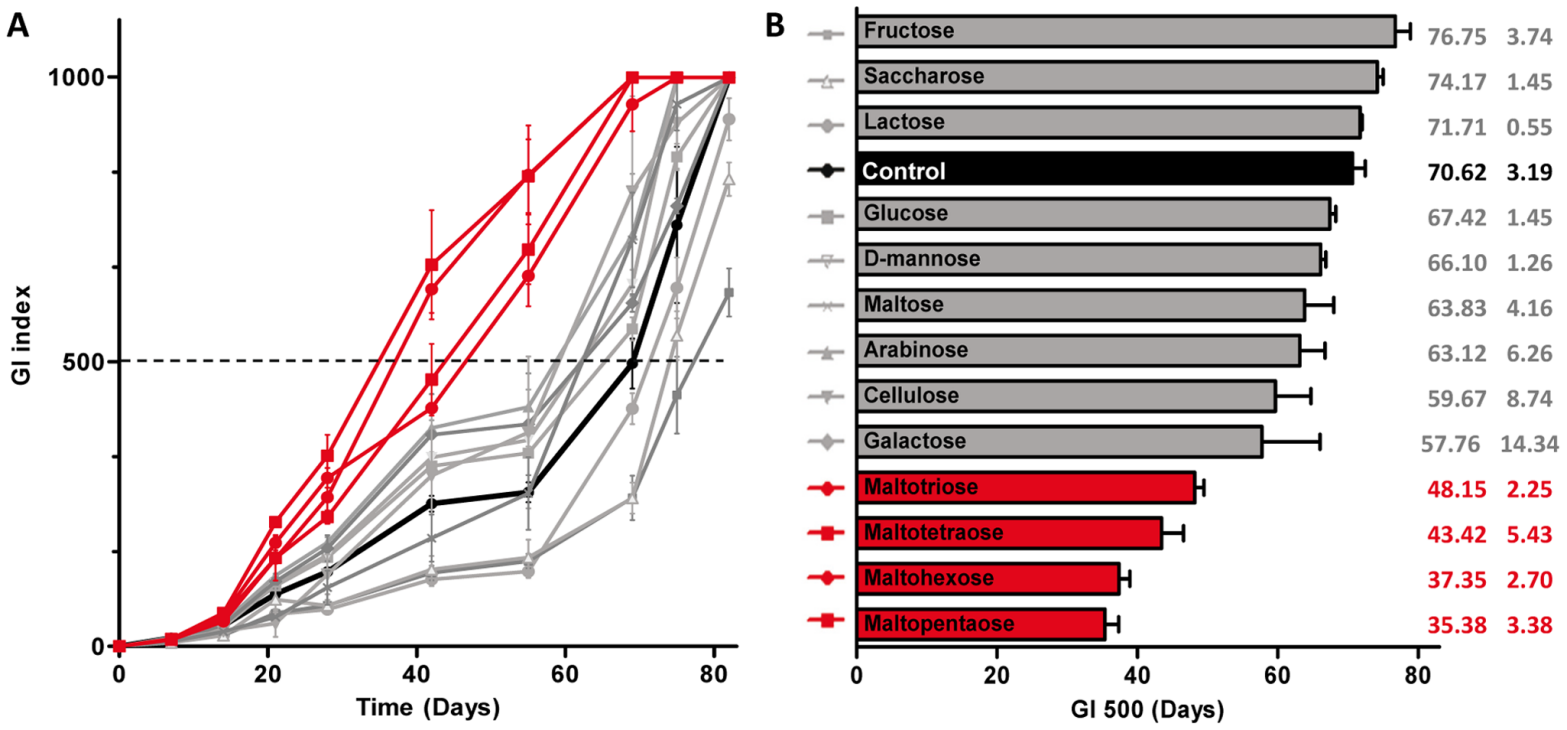
Effects of defined carbohydrates on *M. ulcerans* growth. **A-** Growth curves of *M. ulcerans* 1615 strain grown in MGIT medium containing carbohydrate. The growth has been monitored with the BACTEC system. The dotted line corresponds to the Growth Index (GI) 500. **B-** Growth Index 500 corresponding to the growth curves from A. The control medium is indicated in black. The conditions in which *M. ulcerans* growth is significantly stimulated are indicated in red (*p*<0.001).

We have previously shown that crude extracts from green algae, *Rhizoclonium* sp. and *Hydrodictyon reticulatum*, halved the doubling time of *M. ulcerans* [22]. In order to identify the organic compounds secreted by these plants involved in this stimulation mechanism, we screened a variety of carbohydrates for their capacity to stimulate 1615 *M. ulcerans* strain in liquid broth. The bacterial growth was monitored with the BACTEC system with addition of various carbohydrates (Figure 1A). We have chosen several monosaccharides which are retrieved in algae composition like glucose, mannose or galactose [51], [52]. Disaccharides or polysaccharides derived from glucose were also tested. As control, a plant-unrelated disaccharide, lactose was tested. Some carbohydrates stimulated *M. ulcerans* growth and the growth index 500 corresponding to the midpoint of the growth curves was calculated in order to quantify this stimulation (Figure 1B). Indeed, growth index 500 was ranged between 35 to 48 days with addition of maltopentaose, maltohexose, maltotetraose or maltotriose. In the control medium, this index was obtained around 70 days. Interestingly, all stimulating carbohydrates are polymers of maltose, a disaccharide composed of two units of glucose. Surprisingly, cellulose did not stimulate significantly bacterial growth. The difference between cellulose and the other glucose polymers lies on the nature of the O-glucosidic bond. Indeed, in cellulose the glucose units are β1–4-linked while the other polysaccharides derived from maltose have α-glucosidic bonds.

Based on the observation that glucose polymers were able to stimulate *M. ulcerans* growth, we decided to check the stimulating effect of dextrin from maize starch, a polysaccharide with α-glucosidic bonds. We observed a similar stimulation with starch as with maltopentaose (Figure S1). Indeed, the ratio of growth index 500 of the maltopentaose compared to the control medium was 0.50 and the ratio for the starch medium was 0.56, indicating a strong growth stimulation by both media.

### Growth stimulation by glucose polymers is common to various *M. ulcerans* strains

In order to check if this growth stimulation by starch is general to this species, six *M. ulcerans* strains isolated from Buruli ulcer patients have been grown in microMGIT (Mycobacterium Growth Indicator Tubes) tubes containing 7.5% of starch. This starch concentration is the optimal one for growth stimulation determined in our laboratory condition (data not shown). In all cases, the time for bacterial cultures to become positive was significantly highly reduced in presence of starch compared to the regular medium (Table 1). The time gain ranged between 13 to 30 days. These results suggested that the growth stimulation by glucose polymers, especially starch, is not specific to one strain but is general to *M. ulcerans* species.

### Medium-enrichment with glucose induces modification in *M. ulcerans* phenotype on agar plate and cell surface properties

**Figure 2:**
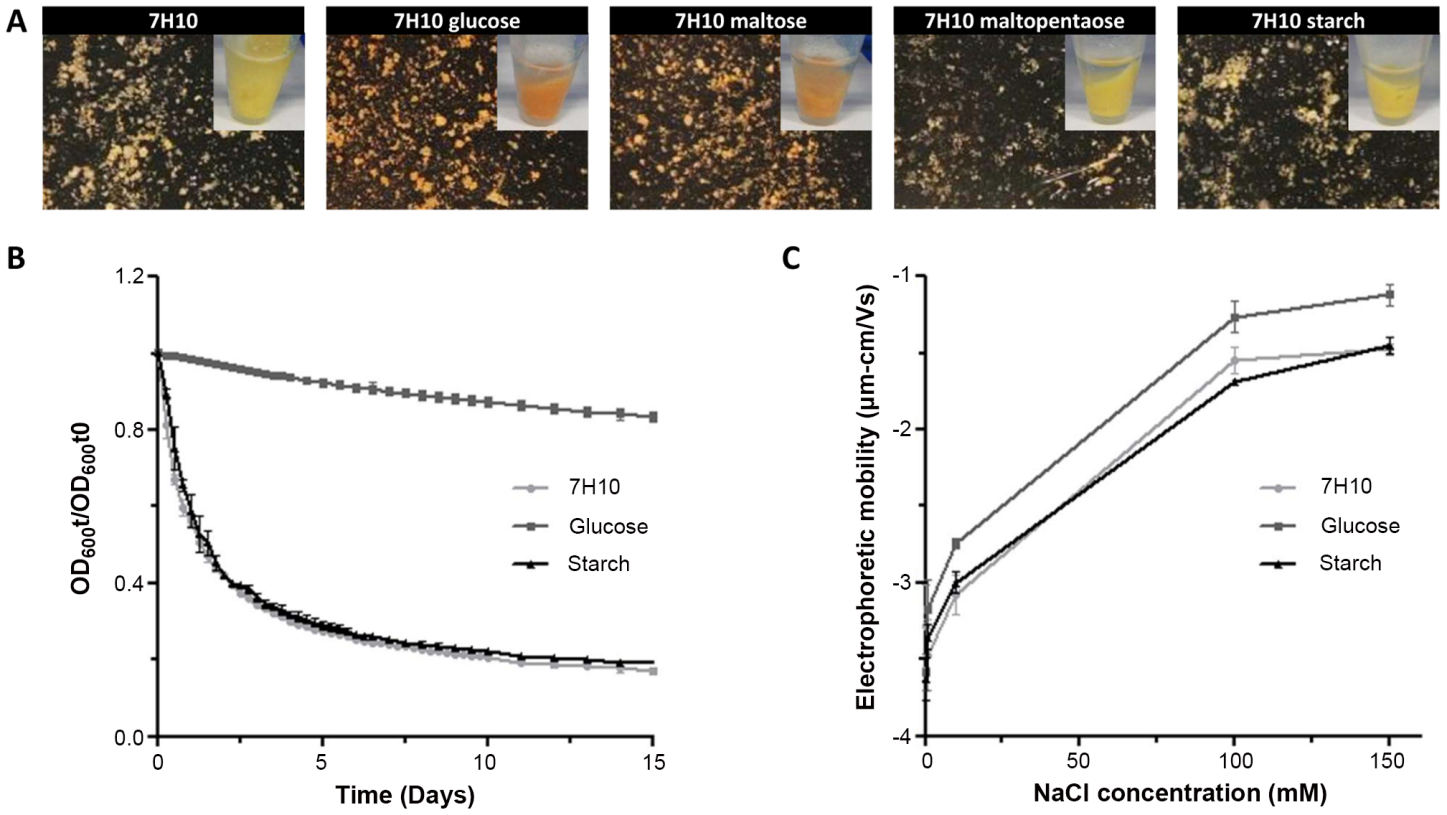
Macroscopic phenotypes of *M. ulcerans* 1615 strain grown on carbohydrates-containing 7H10 media. **A-** Phenotypes on 7H10 plates and picture of the corresponding pellets. **B-** Quantification of aggregative abilities of *M. ulcerans* 1615 strain grown on 7H10 medium containing 7.5% of glucose or starch by measuring the decrease in OD_600_ upon sedimentation of bacterial aggregates in static liquid cultures. Optical density has been measured and compared to initial optical density (t = 0). Relative OD_600_ is indicated. **C-** Electrophoretic mobility of *M. ulcerans* 1615 strain grown on 7H10 medium containing 7.5% of glucose or starch, measured according to NaCl concentration in the suspending medium at constant pH.

In order to bring out some carbohydrates-linked modifications in the mycobacterial metabolism, large cultures of *M. ulcerans* were performed on 7H10 agar plates enriched with 7.5% maltopentaose or starch to obtain an important biomass. As controls, media complemented with monosaccharides (glucose and maltose) were added. Unexpectedly, we observed variations in *M. ulcerans* phenotype depending on culture media. Indeed, *M. ulcerans* colonies appeared orange on glucose- and maltose-containing plate (Figure 2A). For maltopentaose and starch-rich media, wild-type yellow phenotypes, likely due to mycolactone production [9], were noticed. Phenotypical changes observed with 7.5% glucose or maltose-enriched medium could be explained by variation of toxin production, as mycolactone-negative *M. ulcerans* strains have a white phenotype on agar plate [9], or could result from other variations in cell wall composition.

Another macroscopic difference was rapidly noticed by collecting bacterial colonies from plates. Indeed, cultures from glucose plate showed a reduced aggregation. Aggregative ability was thus quantified by measuring the decrease in optical density (OD) that occurs in non-agitated liquid cultures upon bacterial sedimentation [43]. Aggregates from *M. ulcerans* grown on 7H10 plate almost completely and rapidly sedimented (Figure 2B). On the contrary, bacteria grown on glucose-containing plate displayed a dramatically decreased aggregation (12.8%±2.3 of bacterial aggregates were sedimented after 10 minutes against 79.3%±1.0 for the 7H10 medium). The aggregative ability of *M. ulcerans* grown on starch-containing plate was found to be indistinguishable from that of 7H10 condition. The difference in aggregative ability could reflect variation in cell wall properties (hydrophobicity or charge).

To investigate the possibility of modification in the mycobacterial cell wall hydrophobicity, adherence to hexadecane, an apolar solvent, was checked [44], [53]. In three cases, high affinity to hexadecane was measured revealing elevated hydrophobic character. However, the affinity to hexadecane of *M. ulcerans* grown on glucose-supplemented medium was estimated to 86.72%±2.98 against 94.45%±1.52 and 93.52%±0.95 for bacteria grown on 7H10 and 7H10starch, respectively. These differences are statistically significant (*p*-values<0.05). The bacilli grown with glucose were thus slightly less hydrophobic compared to bacteria grown on 7H10 or 7H10starch.

We then examined bacterial surface charge, which is a reflection of the moieties exposed on the outer envelope. The electrophoretic mobility (EM), which is directly proportional to the zeta potential, which in turn reflects the overall charge that a particle acquires, was measured for *M. ulcerans* grown in the three conditions. The electrophoretic mobility of particles was measured at different ionic strength by changing the concentration of NaCl. The three samples exhibited a negative EM for all the ionic strengths studied (Figure 2C). However, the absolute values of EM were significantly lower for 7H10 or 7H10starch bacteria than for 7H10glucose, showing that the 7H10glucose bacteria are less electronegatively charged.

### Culturing of *M. ulcerans* in medium supplemented with different glucose polymers does not induce visible morphological changes

The phenotypical data on agar plate and cell surface properties seem to indicate that the cell wall composition was modified when mycobacteria were cultured in medium supplemented with glucose. We, therefore, sought out to establish whether such modifications induced cell wall alterations or other morphological changes. To this aim, *M. ulcerans* was cultured in medium supplemented with different sugars after which bacteria were fixed and processed for conventional transmission electron microscopy (TEM).

**Figure 3:**
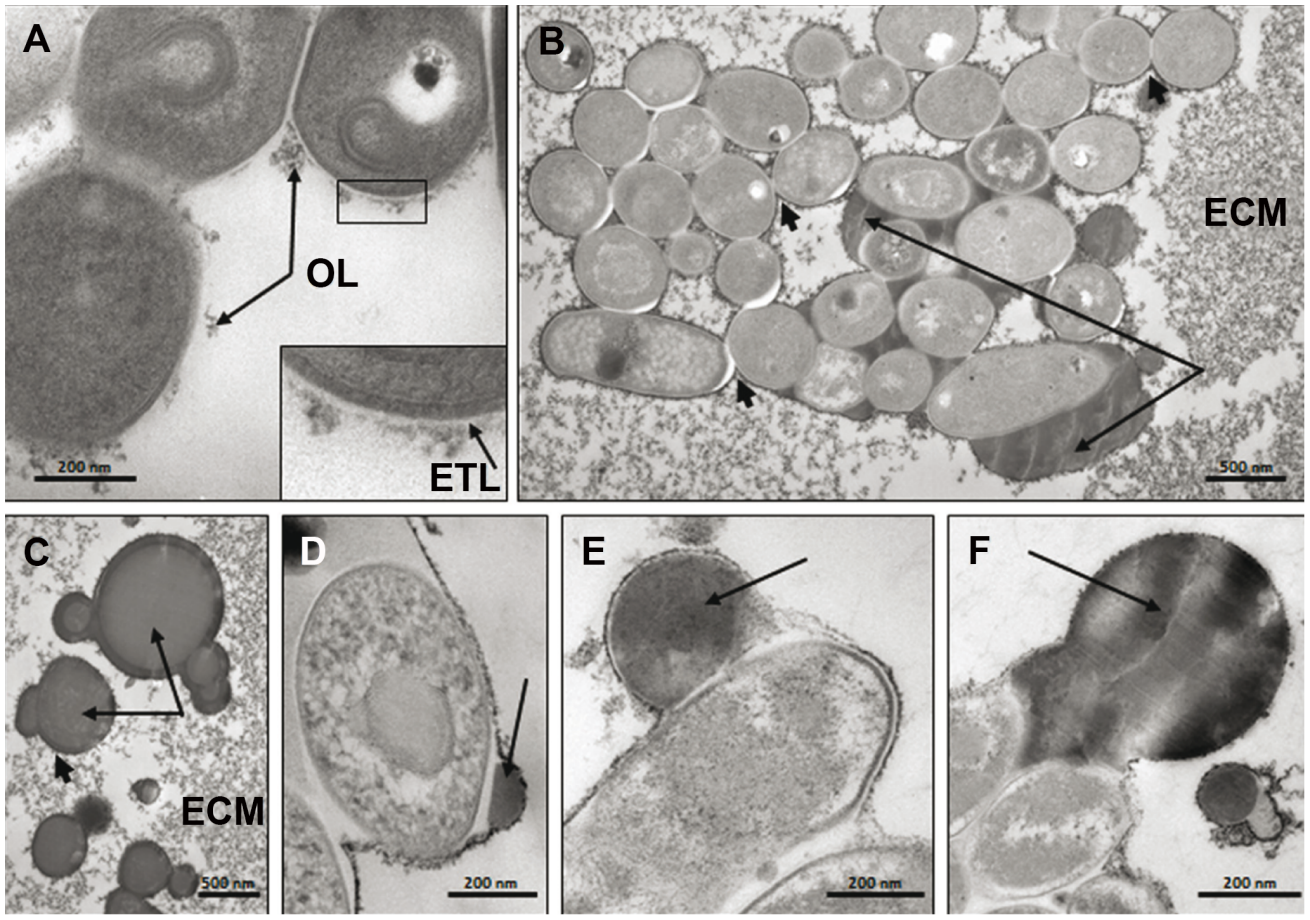
Morphological features of *M. ulcerans* are not modified by carbohydrates added to the medium. *M. ulcerans* strain 1615 was grown in MGIT medium alone or supplemented with 7.5% glucose, maltose, maltopentaose or starch, fixed in the absence (**A**) or presence (**B–F**) of 0.1% Malachite green and processed for transmission electron microscopy (TEM). **A-** Cell wall ultrastructure of bacilli grown in normal medium. Note the presence of the thin electron translucent layer (ETL) and the dense outer layer (OL). Inserts show enlarged view of cell wall (rectangle). **B-** Morphological appearance of a bacterial clump surrounded by OL (small arrows). Lipids (long arrows) fill the space between bacilli. Note the thick extracellular matrix (ECM). **C-** Morphological appearance of lipid-rich vesicles (long arrows) surrounded by OL (small arrow) in the ECM. **D, E, F-** Formation of lipid-rich blebs at the bacterial surface (arrows).

In normal medium, *M. ulcerans* is most frequently clumped in more or less large aggregates although isolated bacilli can also be found. Bacilli displayed a typical cytoplasmic membrane and cell wall, including a thin electron translucent layer beyond the peptidoglycan layer and an outermost electron-dense layer (OL) reminiscent of the one described by Zuber *et al.* [54] (Figure 3A). In the case of clumped bacteria, the OL surrounded the entire clump rather than the individual bacilli (Figure 3B). Another striking feature of *in vitro* growing *M. ulcerans* deserves special mention, viz. the presence of a large extra-cellular matrix (ECM) with which bacteria seemed to establish an intimate contact (Figure 3B). The origin of the ECM is unknown but could result from shedding of the dense layer surrounding the clumps. The ECM usually appeared as densely-packed fibrillar material but it also displayed electron translucent zones that could be lipid-enriched.

In order to detect the presence of lipids in the ECM, bacilli were fixed in presence of 0.1% Green Malachite. This dye stabilizes and stains lipids that become electron-dense and hence easy to visualize under the TEM [45]. With this method, many lipid-rich vesicles were found in the ECM. They consisted of a core of more or less densely packed lipids surrounded by a layer of very dense fibrous material resembling the dense OL surrounding the mycobacterial cell wall (Figure 3C). The presence of this layer suggests that lipid-rich vesicles might originate from bacilli. A closer examination of bacilli indeed showed that lipids could be seen first between bacilli within a clump (Figure 3B) and then concentrated in discrete and increasingly larger blebs at the mycobacterial surface, where they were surrounded by the dense OL (Figure 3D–F).

Culturing of *M. ulcerans* in presence of starch, maltopentaose, maltose or glucose did not affect growth of mycobacteria in clumps or as individual bacilli nor induce visible alterations of the cell wall ultrastructure. Likewise, formation of the ECM and production of lipid-rich vesicles as well as the ultrastructural appearance of both structures were not affected by the presence of carbohydrates. TEM does not allow, however, to determine whether the nature and/or abundance of lipids in these vesicles varies according to the carbohydrate added to the medium.

### Effect of glucose or maltose-enriched media on the glycolipid composition of *M. ulcerans*

In an initial attempt to analyze changes in the cell envelope composition of *M. ulcerans* grown in the presence of different carbon sources, we focused on the lipoglycans, lipomannan (LM) and lipoarabinomannan (LAM) and the mycolyl-arabinogalactan-peptidoglycan (mAGP) complex constituting the cell wall core. These essential components insulate the bacteria from its environment and play diverse roles in the bacteria–host interactions [55]. The SDS-PAGE analysis revealed the presence of comparable amounts of LM and LAM in the various cell extracts (Figure S2A). Likewise, the monosaccharide composition of mAGP complex isolated from the different bacterial cultures was compared and no significant differences were found (Figure S2B).

**Figure 4:**
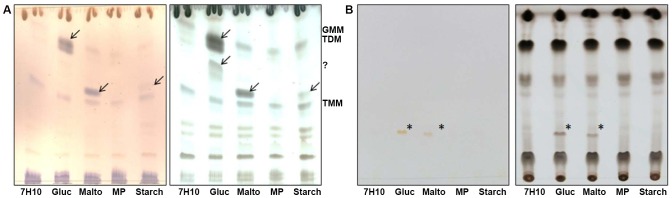
Total lipids analysis of *M. ulcerans*. **A-** Lipids from *M. ulcerans* were separated by TLC using CHCl_3_/CH_3_OH/H_2_O [20∶4∶0.5, by volume] as the solvent system. Lipids were revealed with alpha-naphthol (left panel) or cupric sulfate (right panel) followed by heating. **B-** Lipids from *M. ulcerans* were separated by TLC using CHCl_3_/CH_3_OH [98∶2, by volume] as the solvent system. TLC are shown before (left panel) and after revelation with cupric sulfate and heating (right panel). Arrows highlight new lipid forms in the bacteria grown in 7.5% glucose and maltose-enriched media compared to regular 7H10. Gluc, 7H10 glucose; Malto, 7H10 maltose; MP, 7H10 maltopentaose; starch, 7H10 starch. TDM, trehalose dimycolate; TMM, trehalose monomycolate; GMM, glucose monomycolate; MMM, maltose monomycolate. The compound migrating between TMM and TDM in glucose-grown bacteria does not stain with alpha-naphthol indicating that it is not a glycolipid. Its identity was not determined here.

To analyze potential changes in the lipid composition of *M. ulcerans* grown in the presence of various sugars, total lipids were extracted and analyzed by TLC using solvent systems of different polarities. No significant changes were observed at the level of phosphatidylinositol mannosides (PIM), phospholipids, phthiodiolone diphthioceranates and phenolphthiodiolone diphthioceranates (DIP) and triglycerides (TAG) (Figures 4A and S2). However, new glycolipid forms migrating in the region of TDM (trehalose dimycolate) and TMM (trehalose monomycolate) were observed in the bacteria grown in 7.5% glucose and maltose-enriched media (Figure 4A). The compound produced by bacteria grown in 7.5% glucose-enriched medium and migrating at the level of TDM was identified by liquid chromatography-mass spectrometry as glucose monomycolate (GMM) (Figure S4). The glycolipid migrating above TMM in the bacteria grown in maltose- or, to a lesser extent, 7.5% starch-enriched media displayed the expected mass for either maltose monomycolate or trehalose-monomycolate (the compounds share the exact same mass) (Figure S5) and was tentatively identified as maltose monomycolate based on its TLC migration properties.

### Toxin synthesis is drastically decreased by glucose, maltose and maltopentaose

**Figure 5:**
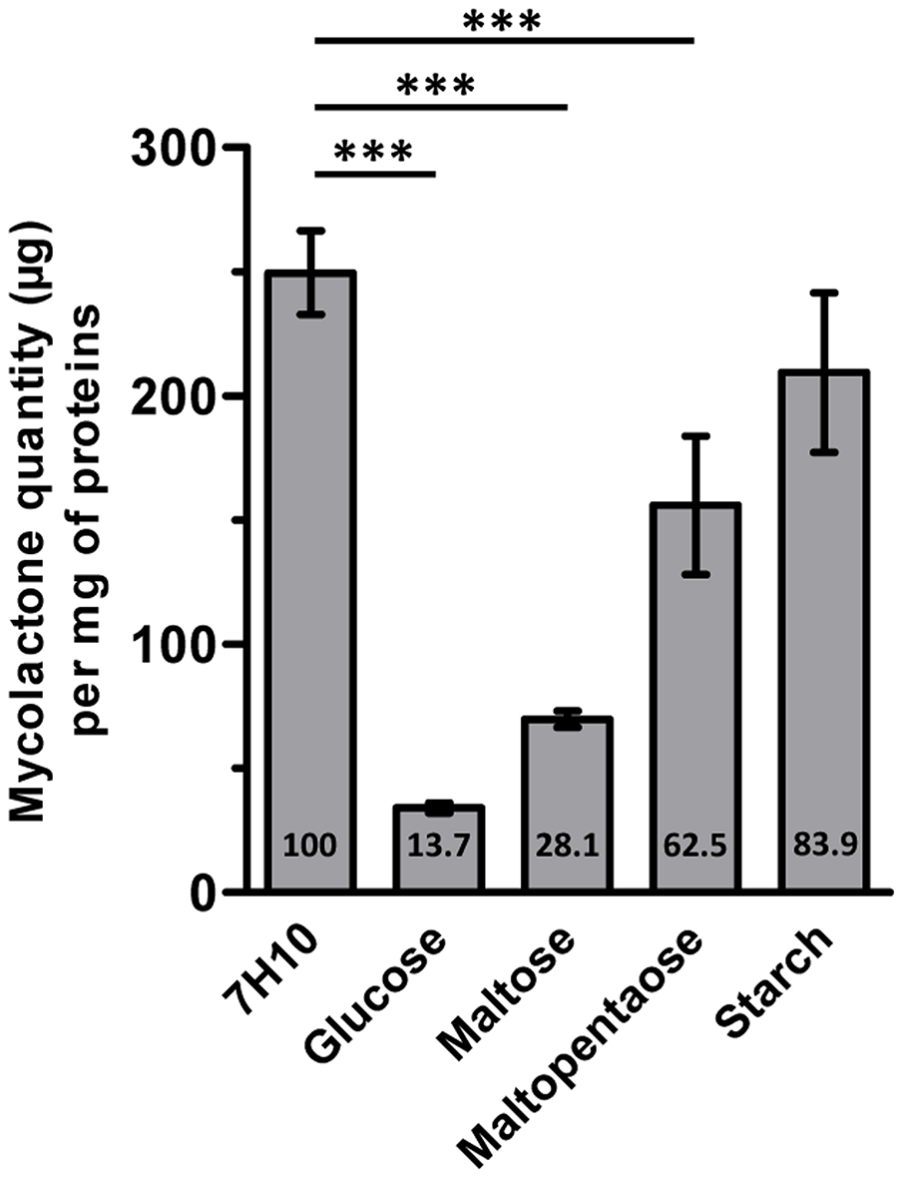
Mycolactone quantification by HPLC from *M. ulcerans*. Relative quantities have been calculated after normalization to the proteins quantities in each sample. The percentages compared to the 7H10 medium are indicated in the chart. *** *p*<0.001. ** *p*<0.05.

In order to check the toxin production in bacteria grown on carbohydrate-containing media, we performed HPLC quantification. As shown in Figure 5, bacteria cultivated on 7.5% glucose-enriched medium displayed a drastically reduced mycolactone content compared to bacteria cultivated on 7H10. The amount of mycolactone produced by the bacteria on glucose-rich medium was only 13.7% of that produced in 7H10 medium. A similar albeit less marked trend was observed in the bacteria grown on 7.5% maltose- or maltopentaose-enriched medium. In contrast, no statistical difference was observed in toxin production between bacteria grown on 7H10 and starch-supplemented 7H10 media. These results indicate a down-regulation of mycolactone production by some carbohydrates.

### Toxin regulation by carbohydrates is not under transcriptional or translational control

**Figure 6:**
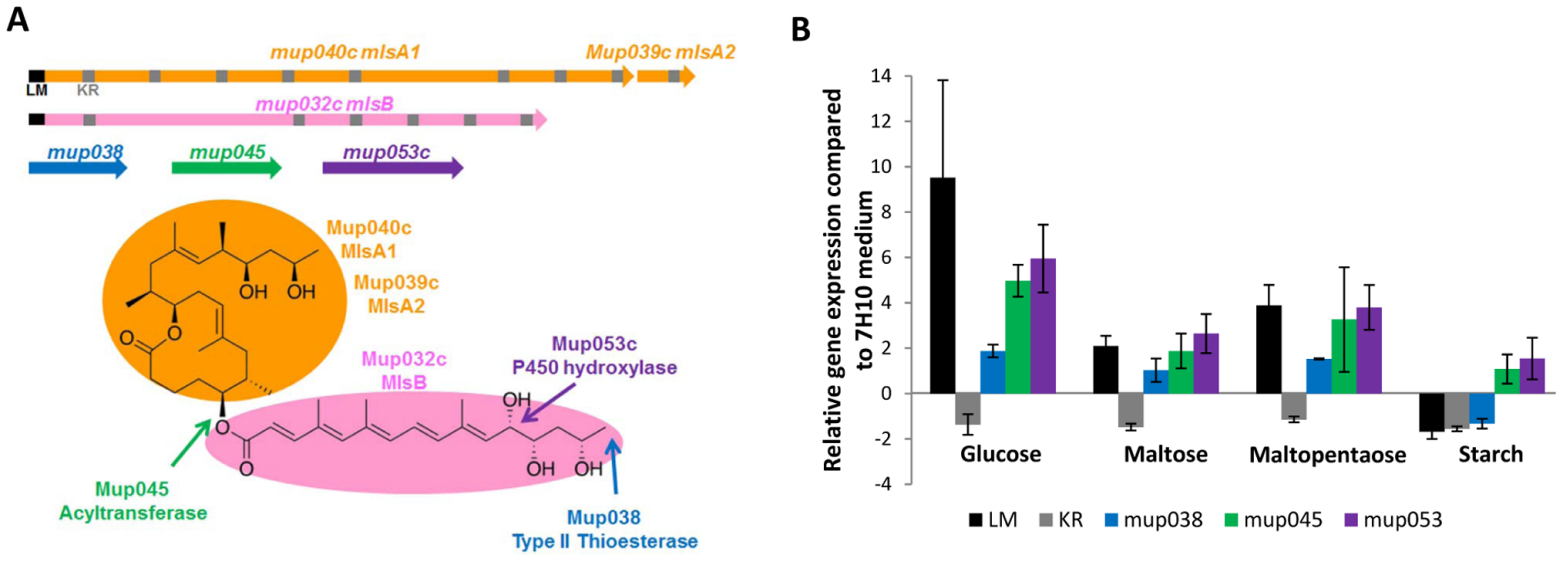
Effect of carbohydrates on mycolactone biosynthesis gene expression in *M. ulcerans*. **A-** Schematic representation of mycolactone biosynthesis genes and representation of their function on the mycolactone structure [9], [10]. The six genes coding for proteins involved in mycolactone synthesis are located on the giant plasmid named pMUM001. The genes *mlsA1* (50,973 bp) and *mlsA2* (7,233 bp) encode modular type I polyketide synthase (PKS) required for the biosynthesis of the mycolactone core (highlighted in orange). The side chain enzyme is encoded by *mlsB* gene (42,393 bp) in pink. The *mls* genes encode 11 different functional domains that are repeated along the gene. Among these modules, the two load modules (LM, in black) are present at 5′end of *mlsA1* and *mlsB* genes and 15 ketoreductase (KR, in grey) are present. There are also three genes coding for potential polyketide-modifying enzymes, including a P450 hydroxylase (*mup053*, in purple), probably responsible for hydroxylation at carbon 12 of the side chain, a potential FabH-like type III ketosynthases (KS) with an acyltransferase activity that might catalyze the C–O bond between the mycolactone core and side-chain (*mup045*, in green) and a putative type II thioesterase (*mup038*, in blue) that may be required for removal of short acyl chains from the PKS loading modules, arising by aberrant decarboxylation. **B-** Analysis of mycolactone-associated gene expression in *M. ulcerans* grown on various 7.5% sugar-enriched medium by qRT-PCR. Gene expression has been normalized to the constitutively expressed housekeeping gene *ppk* encoding the polyphosphate kinase. Relative genes expression is expressed as fold-changes relative to the 7H10 medium. Data are presented as the mean and SD of at least three biological repeats. LM, load module domain.

To understand the regulation process of mycolactone, the transcript level of genes required in mycolactone biosynthesis has been analyzed by RT-qPCR. The expression of the *mls* genes required for the mycolactone core and side chain has been analyzed (LM and KR domains) as well as the expression of the three accessory genes (*mup038*, *mup045*, *mup053c*) (Figure 6A). In Figure 6B, gene expressions are expressed as fold-changes relative to the 7H10 medium. In 7.5% glucose-enriched condition, *mup038*, *mup045*, *mup053c* and *mls* (LM module) genes were overexpressed compared to the 7H10 medium (fold-changes of 1.87±0.28, 4.97±0.70, 5.95±1.50 and 9.51±4.30, respectively) (Figure 6B). In maltose and maltopentaose media, *mup053c* and *mls* (LM module) genes were also overexpressed (respective fold-changes of 2.09±0.45 and 2.64±0.86 for maltose, 3.88±0.91 and 3.79±0.99 for maltopentaose). Thus, paradoxically, the transcript level of the mycolactone biosynthesis genes seemed to be up-regulated under conditions where mycolactone content was found to be reduced.

Quantitative proteomic approach by NanoLC MS/MS was utilized to determine the effect of carbohydrates on bacterial metabolism. The relative abundance of 1076 proteins (24.67% of total *M. ulcerans* proteins) has been determined and ratios compared to the 7H10 medium were calculated (Table S2). Among the identified proteins, 822 (76.39%) were not regulated in the five tested conditions. In the 254 remaining proteins, we selected the proteins quantified with at least two peptides and with a *p-value*<0.05. This analysis defined 135 carbohydrate-response proteins of which 8 proteins were induced and 8 were repressed upon growth on 7.5% glucose, maltose, maltopentaose or starch-enriched medium. Depending on growth condition, the number of regulated proteins could vary from 27 to 111 (Figure S6A, B and C). The repartition in functional class of proteins did not change in various conditions (Figure S7A and B).

Three proteins involved in mycolactone biosynthesis have been shown to be overproduced with glucose or maltose and, to a lesser extent, in 7.5% maltopentaose- or starch-enriched medium (Table 2). Indeed, Mup053c is overproduced in 7.5% glucose and maltose-enriched media with ratios of 7.67 and 3.26 respectively. For Mup045, an overproduction was observed in all conditions. Proteomic data thus support the transcriptomic data and confirm the increased production of enzymes required for mycolactone synthesis: Mup053 (in glucose and maltose media) and Mup045 (in glucose, maltose and maltopentaose media). Regarding the *mlsA* genes, the overexpression of the LM module in glucose medium was also confirmed by proteomics data indicating an overproduction of MlsA1 protein by a factor of 3.81. These results indicated that mycolactone biosynthesis machinery seems to be overproduced in glucose or maltose-supplemented medium, while the production of its metabolite is reduced in both growth conditions.

**Table 2:** Effect of carbohydrates on the production of some enzymes involved in mycolactone biosynthesis.

				GLUCOSE		MALTOSE		MALTOPENTAOSE		STARCH	
NCBI Accession Number	BuruList Number	Gene name	Gene function	ratio	*p*-value	ratio	*p*-value	ratio	*p*-value	ratio	*p*-value
YP_025561.1	MUP039c	*mlsA2*	Type I modular polyketide synthase	1,757	3,76E-04	1,692	2,09E-04	1,684	8,19E-05	1,587	6,09E-03
YP_025562.1	MUP040c	*mlsA1*	Type I modular polyketide synthase	3,814	5,69E-05	1,500	3,32E-02	2,003	1,19E-02	1,565	2,29E-02
YP_025567.1	MUP045		Beta-ketoacyl synthase-like protein	4,374	3,44E-05	3,057	7,85E-04	2,712	4,85E-04	2,499	1,49E-03
YP_025575.1	MUP053c	*cyp150*	Cytochrome p450 150 cyp150	7,667	1,73E-05	3,256	3,34E-05	1,448	1,32E-03	1,551	1,69E-03

Differentially expressed proteins of *M. ulcerans* grown on 7H10 enriched with 7.5% of various carbohydrates.

### Slight variation in antigens production depending on growth condition

To further check if medium composition induced modification of *M. ulcerans* antigens production, sera from Buruli ulcer patients were screened by ELISA for reactive antibodies against proteins extract prepared from *M. ulcerans* grown in various conditions. Compared to the reaction against a lysate from *M. ulcerans* grown on 7H10 (mean of 0.308), the immune sera reacted significantly lower (mean of 0.225) against a lysate from *M. ulcerans* grown in 7.5% glucose-enriched medium (*p*<0.001), indicating a difference in antigens production between these two culture conditions (Figure S8). In contrast, the reactivity obtained with a lysate of *M. ulcerans* grown on starch-supplemented medium (0.355) was significantly higher than the one for bacteria grown on 7H10 (*p*<0.05). Unfortunately, qualitative analysis by Western blot did not lead to the identification of putative antigens whose production would be regulated depending on culture conditions (data not shown). In the proteomic analysis, it could be noticed that the secreted antigen 85-A FbpA is up-regulated four times in glucose or maltose conditions compared to other conditions (7H10 complemented or not with maltopentaose or starch) (Table S2), meaning that variation in antigens expression occurs depending on experimental growth conditions. More accurate technics like partial fractionation of the bacterial extract or bidimensional analyses are needed to characterize the antigens detected by the antiserum.

### Impact of growth condition on mammalian host colonization

To determine whether toxin down-regulation affects bacterial ability to colonize its host, mice have been inoculated in the tail by 10^3^ bacilli grown on 7H10 or 7.5% glucose- or starch-enriched medium. The inoculation site for bacterial load was examined weekly and the time of onset of clinical symptoms and symptom severity were noticed. At day 150 post-infection, all mice (7/7) inoculated with bacteria grown on 7H10 or 7.5% starch-enriched medium developed ulceration. On the other side, for glucose-cultured bacteria, only four mice displayed similar lesions, which is significantly different (*P* = 0.07 in a Fisher's exact test). These results suggest that culture on 7.5% glucose-enriched medium seems to have an impact on lesions development and thus probably on host colonization. We are aware of the limitation of our data because of the small number of mice, nevertheless, these results are in accordance with the major role of mycolactone in host colonization like water bugs [30], [31]or mammalian host [13], [56]. Furthermore, our proteomic data indicated that bacteria cultured in 7.5% glucose-enriched medium overproduced some antigens like the secreted antigen 85-A FbpA (Table S2). This may induce a better recognition by the host immune system.

### Decrease in toxin synthesis correlates with an increase in mycobactin production

**Figure 7:**
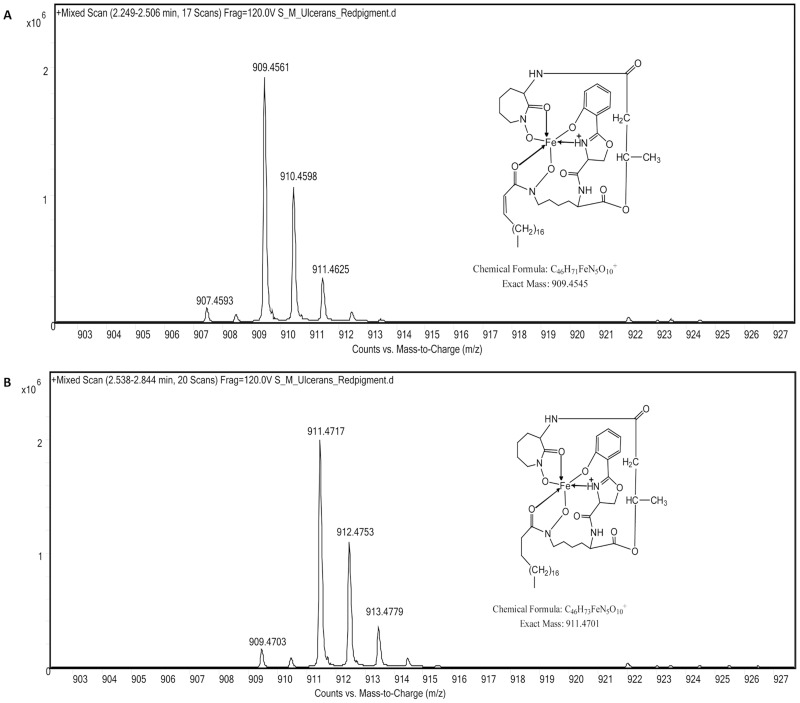
Identification of mycobactins. LC/MS analysis (in positive mode) of the orange compound. Shown are the ion traces at *m/z* 909.45 (A) and at *m/z* 911.47 (B) which are diagnostic for mycobactins differing by one unsaturation.

The orange phenotype of bacteria grown on 7.5% glucose- or maltose-enriched medium was not due to variation of mycolactone production but to the presence of an orange compound extracted with the total lipids from *M. ulcerans* (Figure 4B). This compound was purified by preparative TLC and identified upon LC/MS analysis as mycobactins (Figure 7A–B) [50].

From the quantitative proteomic analysis by nanoLC MS/MS, it appeared that three Mbt proteins encoding enzymes required for the assembly of mycobactin (MbtB, MbtE and MbtG) were overproduced in glucose or maltose (Table S2). MbtE was found to be overproduced by 80.16 and 28.38 in those media. For MbtG protein, no ratio could be calculated since the protein was not detected in 7H10 medium enriched or not with 7.5% maltopentaose or starch. Two other proteins, Mul_3635 and Mul_3636, whose genes are located in the *mbt* locus, were overproduced. Finally, four proteins involved in mycobactin regulation or export (IdeR, IrtA, EsxG and EsxH, MmpS5 and MmpL5) were found to be down- or up-regulated in glucose or maltose conditions (Table S2).

## Discussion

### Stimulation of *M. ulcerans* growth by glucose polymers

Based on previous studies demonstrating the stimulating effect of aquatic plant extracts on *in vitro M. ulcerans* growth [22], we decided to identify the carbohydrates involved in this phenomenon. Our results showed that addition of glucose polymers in Middlebrook 7H12B medium stimulates *M. ulcerans* growth. The growth of *M. ulcerans* thus depends on carbohydrates found in plants. The growth analysis of various clinical *M. ulcerans* strains from three Buruli ulcer endemic regions revealed a common stimulation by starch. The delay for the detection of positive culture was indeed highly reduced for all tested strains. It is thus possible to consider starch, an inexpensive product, as a complement in culture medium to enhance *M. ulcerans* growth. It is noteworthy that Lowenstein-Jensen medium, used for diagnostic labs, contains potato starch (18.75 g/L) whereas glucose (2.0 g/L) is the carbon source in MGIT medium. The addition of starch (7.5%) in MGIT medium would be of particular interest to facilitate the isolation of *M. ulcerans* from the environment. The improvement of *M. ulcerans in vitro* culture will be very useful for genetic manipulation of this slow-growing mycobacterium as it will reduce time to obtain transformants or recombinant clones.

### Down-regulation of mycolactone synthesis in medium enriched in glucose polymers

Our experiments on carbohydrates-dependent growth have led to the identification of growth conditions under which the mycobacterial toxin is down-regulated. Indeed, the mycolactone production was reduced on 7.5% maltopentaose-, maltose- and glucose-enriched medium from 40 to 85%. To our best knowledge, this is the first time that such a regulation is described. By electron microscopy, vesicles have been observed in all tested culture conditions. Smaller vesicles produced by *M. ulcerans* have been already observed by scanning electron microscopy and were shown to be the main reservoir of the mycobacterial toxin [13]. Our observations suggest that ECM and vesicles productions are not dependent on mycolactone biosynthesis by *M. ulcerans*. This is in agreement with previous isolation of ECM and vesicles from *mup045* mutant which does not produce mycolactone [13]. It was also shown that ECM is undetectable in presence of algal extracts [13]. The regulation of toxin production by plant carbohydrates leads to the hypothesis that mycolactone production is probably regulated by environmental signals such as compounds produced by aquatic plants.

The decrease in mycolactone production was not due to a defect in the production of the enzymes required for its biosynthesis. In fact the toxin enzymatic machinery was even overproduced in 7.5% glucose-enriched medium according to the quantitative proteomic study corroborated by the quantification of the transcriptional level of the corresponding genes. Different hypothesis could explain the toxin regulation mechanism. (i) One may hypothesize that a post-translational modification is involved in the regulation of the activity of enzymes involved in mycolactone biosynthesis. (ii) It is also possible that toxin degradation occurs. (iii) Another explanation could reside in the existence of a membrane-anchored enzymatic megacomplex required for mycolactone biosynthesis, as previously shown for other polyketide metabolites from different species [57], [58], [59]. In the case of mycolactone, the MlsA1 and MlsB, as well as Mup053c have been identified in membrane fraction or in vesicles [13], [60]. It could be hypothesize that a mycolactone biosynthetic megacomplex exists and is disassembled due to a repression of some proteins involved in the complex scaffold. (iv) A limitation of substrate for the synthesis of mycolactone could also take place. The up-regulation of the toxin enzymatic machinery could take place to counteract the toxin decrease.

### Decrease in toxin synthesis is linked to iron uptake up-regulation

From an interesting point of view, the decrease in mycolactone production is correlated with the production of the mycobacterial siderophore, the mycobactin. Mycobactins are high-affinity iron-binding molecules and the principle iron acquisition molecules. Iron is an indispensable nutrient for almost all organisms. It is required for the activity of enzymes that are involved in vital cellular functions ranging from respiration to DNA replication [61]. Our results demonstrated that *M. ulcerans* is able to produce this siderophore and confirmed a bioinformatical work showing conservation of *mbt* genes encoding enzymes involved in mycobactin biosynthesis in *M. ulcerans* [62]. We showed that some Mbt enzymes are overproduced in 7.5% glucose-enriched medium. The repression of IdeR, the iron-dependent negative regulator, fits this observation. In addition, other proteins involved in iron metabolism are overproduced. This is the case for IrtA (Mul_3902), an iron regulated transporters of siderophores, Esx-3 encoding EsxG (Mul_1209) and EsxH (Mul_1210), a specialized secretion system required for mycobactin-mediated iron acquisition and for survival during infection, and MmpS5/MmpL5 required for siderophore export (Table S2) [63], [64], [65], [66]. The latest proteins were previously shown to be IdeR-independent and iron-repressed [67]. It is likely that the export machineries of mycolactone and mycobactins act as scaffolds for their respective biosynthetic machineries. Coupled elongation and export machineries are indeed not uncommon in the biogenesis of mycobacterial polyketide-derived lipids and abolition of their biosynthesis caused by defects in the expression of the export machinery or failure of the biosynthetic enzymes to interact with the transporters have been reported in the case of phthiocerol dimycocerosates and glycopeptidolipids [57], [58]. If one hypothesizes that mycolactone and mycobactins and their respective biosynthetic complexes compete for the same translocation machinery (like MmpL5/MmpS5, recently shown to be required for mycobactin export in *M. tuberculosis* [66]), then this may explain the disappearance of mycolactone when mycobactin production is induced.

It was shown that production of siderophores is a major point for the regulation of iron uptake. These molecules are produced exclusively under iron limitation when the bacterium needs them, and increasing the concentration of available iron turns off their synthesis [61]. Intriguingly, it appears from our experimental work that iron metabolism is activated in glucose or maltose-rich medium, without the influence of the iron concentration which has never been modified. This suggests that the iron regulon is activated by other signal that iron concentration. It may be part of another regulon. It could be noticed that in the proteomic analysis, no sigma factors were found to be regulated in glucose-rich medium.

### Bacterial adaptation to ecological niches: Balance between virulence and persistence in the environment

We have thus shown that siderophore overproduction is correlated to the toxin down-regulation. Therefore, one question emerges: are their production biochemically linked or are they oppositely regulated by the same environmental signals? Indeed, we have shown that some carbohydrates like maltopentaose or starch, both derived from maltose, are able to stimulate *M. ulcerans* growth. On the opposite, some carbohydrates are able to reduce toxin production and to induce iron metabolism. These observations suggest a regulation of *M. ulcerans* metabolism by environmental signals. In some conditions, high multiplicity and toxin production could confer capacity to *M. ulcerans* for host colonization. In other conditions, *M. ulcerans* adapts its metabolism by reducing mycolactone production and increasing iron metabolism. Whereas not required for planktonic growth in *M. smegmatis*, iron is essential for biofilm development [68]. We could speculate that our *in vitro* culture condition mimics environmental conditions in which *M. ulcerans* does not need to produce toxin to survive and activate the iron pathway to colonize specific niches in their environment. One can also ask what the role of iron concentrations in toxin metabolism is. Indeed, as mycobactine production is stimulated in low iron concentration, its production should be limited in environments rich in iron and the production of mycolactone would be increased if the biosynthesis of these two metabolites is linked. In the infectious cycle of *M. ulcerans*, the high concentration of iron in human blood could induce reduction in siderophore production and increase of toxin production by bacteria from the early to the late stages of infection. This hypothesis could explain the fact that *M. ulcerans* infection occurs by environmental contact such as insect bite and that no human-to-human transmission had been observed. On the other hand, it is possible that the *in vitro* medium enrichment by starch mimics the bacterial environment in cutaneous ulcer.

It has been described that *M. ulcerans* is able to colonize salivary glands of *Naucoris cimicoides* from Naucoridae family [30] and is present in saliva of *Appasus sp.* from Belostomatidae family [29]. It is well known that insect salivary glands contain α-glucosidases and α-amylases that initiate the digestion of carbohydrates. The activity of both type of enzymes in salivary glands of *B. lutarium* (Belostomatidae family) has demonstrated [69]. Their activity leads to high level of glucose. It is thus possible that such glucose concentration could inhibit the mycolactone biosynthesis when *M. ulcerans* colonize this habitat, explaining the absence of tissue damage mediated by the cytotoxic activity of the mycolactone whereas the insect cells are sensitive to the toxin [30].

The study of mycolactone regulation is essential to deepen our knowledge on the mechanisms of *M. ulcerans* virulence. Unfortunately, some tools to quantify this polyketide toxin sorely lacking. Nevertheless, this work is the first step in mycolactone regulation understanding. Indeed, for the first time, we have identified culture conditions influencing its production. Those media enriched with carbohydrates could reflect ideal conditions to study the mechanisms of mycolactone regulation and its role in the ecology of *M. ulcerans*. The understanding of such mechanisms is a key issue for a better comprehension of Buruli ulcer physiopathology and the identification of putative therapeutic targets.

## Supporting Information

Figure S1 **Effects of starch on** ***M. ulcerans*** **growth.** Growth curve of *M. ulcerans* 1615 strain grown in MGIT medium containing starch (in red). The growth has been monitored with the BACTEC system. The control medium is indicated in black.
(TIF)

Figure S2 **Analysis of lipomannan (LM), lipoarabinomannan (LAM) and mycolyl-arabinogalactan-peptidoglycan (mAGP).** **A-** Analysis of LM and LAM from *M. ulcerans* 1615 strain grown on 7.5% carbohydrates-enriched 7H10. Lipoglycans were separated on a 10–20% Tricine gel and visualized by PAS staining. **B-** Monosaccharide composition of the mAGP complex of *M. ulcerans* 1615 strain grown under various conditions. mAGP was subjected to alditol acetate preparation. The mole percentage of each monosaccharide is indicated on the graph and the ratios of arabinose to galactose reflecting the composition of the major cell wall heteropolysaccharide, arabinogalactan, presented in the table. GlcNAc, N-acetyl-D-glucosamine; GalNAc, N-acetylgalactosamine.
(TIF)

Figure S3 **Total lipid analysis of** ***M. ulcerans*** **.** Lipids from *M. ulcerans* were separated by TLC in two different solvent systems. **A-** Petroleum ether: ethyl acetate (98∶2; three developments). **B-** CHCl_3_/CH_3_OH/H_2_O (65∶25∶4). Lipids were revealed with alpha-naphthol (left panel) or cupric sulfate (right panel) followed by heating. Arrows highlight new lipid forms in 7.5% glucose, maltose and starch-enriched media compared to regular 7H10 medium. Gluc, 7H10 glucose; Malto, 7H10 maltose; MP, 7H10 maltopentaose; starch, 7H10 starch. CL, cardiolipin; DIP, phthiodiolone diphthioceranates and phenolphthiodiolone diphthioceranates; PE, phosphatidylethanolamine; PI, phosphatidylinositol; PIM, phosphatidylinositol mannosides; TAG, triacylglycerol; TMM, trehalose monomycolate.
(TIF)

Figure S4 **Identification of glucose monomycolate.** (A) Total ion chromatogram of the LC/MS analysis of the TLC spot identified as GMM. (B) The mass spectrum of the major component of the starred peak in (A) showing a M+Na^+^ ion at m/z 1280.1722 (C_81_H_156_NaO_8_ with a calculated value of *m/z* of 1280.1670) and a M+NH_4_^+^ ion at *m/z* 1275.2146 (C_81_H_156_NaO_8_ with a calculated value of *m/z* of 1275.2139). (C) A structure consistent with the molecular weight data of (B) where the unsaturation is arbitrarily shown as cyclopropyl groups. (D) Total ion chromatogram of the LC/MS analysis of the TLC spot after per-*O*-acetylation. (E) The mass spectrum of the major component of the starred peak in (D) showing a M+Na^+^ ion at *m/z* 1490.2242 and a M+NH_4_^+^ ion at *m/z* 1485.2684. Both ions are consistent with the presence of five acetyl groups (one on the mycolyl hydroxyl group and four on C-1, C-2, C-3, and C-4 of the hexosyl residue thought to be glucose). (F) The structure as in (C) but with the 5 acetyl groups indicated.
(JPG)

Figure S5 **Identification of maltose monomycolate.** (A) Total ion chromatogram of the LC/MS analysis of the TLC spot identified as maltose monomycolate. (B) The mass spectrum of the major component of the starred peak in (A) showing a M+Na^+^ ion at m/z 1456.2387 (C_88_H_168_NaO_13_ with a calculated value of *m/z* of 1456.2377) and also M+Na^+^ ion at *m/z* 1470.2538 (C_89_H_168_NaO_13_ with a calculated value of *m/z* of 1470.2534). (C) A structure consistent with the molecular weight data of (B) where the unsaturation is arbitrarily shown as cyclopropyl groups. (D) Total ion chromatogram of the LC/MS analysis of the TLC spot after per-*O*-acetylation. (E) The mass spectrum of the major component of the starred peak in (D) showing a M+Na^+^ ion at *m/z* 1792.3216 and a M+NH_4_^+^ ion at *m/z* 1787.3639. Both ions are consistent with the presence of 8 acetyl groups (one on the mycolyl hydroxyl group, three on C-2, C-3, and C-4 of the maltose linked to lipid, whereas the other four on C-4 linked maltose residue at C-1, C-2, C-3 and C-6 positions). (F) The predicted structure as in (C) but with the eight acetyl groups indicated. The identity of the di-hexosyl residue was not directly determined but assumed to be maltose based on the TLC migration properties of the glycolipid above TMM.
(TIF)

Figure S6 **Repartition of** ***M. ulcerans*** **regulated proteins grown on 7.5% carbohydrates-enriched 7H10.** **A-** Number of up- or down-regulated proteins in *M. ulcerans* grown on various media. Venn diagram showing the distribution of shared overproduced (**B**) or repressed (**C**) proteins among *M. ulcerans* grown on different media.
(TIF)

Figure S7 **Distribution of functional protein categories of** ***M. ulcerans*** **regulated proteins grown on 7.5% carbohydrates-enriched 7H10.** **A-** Distribution of functional categories of overproduced proteins. **B-** Distribution of functional categories of repressed proteins. The functional category of each protein was determined though BuruList website. They correspond to virulence, detoxification, adaptation; lipid metabolism; information pathways; cell wall and cell processes; PE/PPE; intermediary metabolism and respiration; regulatory proteins; conserved hypotheticals.
(TIF)

Figure S8 ***M. ulcerans*** **antigens production in various conditions.** Human IgG Binding to whole proteins lysate by ELISA Assay. Mean values are indicated by black thick drawbar. Each of the 21 tested sera has its own symbol. *** *p*<0.001. ** *p*<0.05.
(TIF)

Material and Methods S1 **Ethical aspect, alditol acetates of Arabinogalactan (AG), lipoarabinomannan (LAM) and Total cells, proteomic analysis by NanoLC MS/MS, human sera and ELISA analysis and supplementary references.**
(DOC)

Table S1 **Primers used in this study.**
(TIF)

Table S2 **Differentially expressed proteins of** ***M. ulcerans*** **grown on 7H10 enriched with 7.5% of various carbohydrates.** The production of proteins was considered significantly altered with a *p*-value<0.05 in a Student's *t*-test and with 2-fold change compared to regular 7H10 medium.
(XLSM)
