# Supplementary figures and images for: Regulation of Mycolactone, the Mycobacterium ulcerans Toxin, Depends on Nutrient Source
Source: PLoS Negl Trop Dis. 2013 Nov 14;7(11):e2502. doi: 10.1371/journal.pntd.0002502 (PMC3828164; doi:10.1371/journal.pntd.0002502)

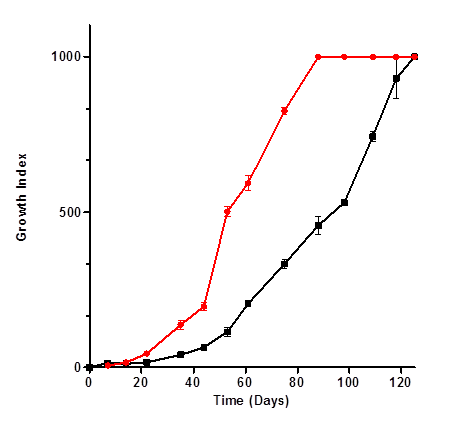

Supplement: Figure S1 — Effects of starch on M. ulcerans growth. Growth curve of M. ulcerans 1615 strain grown in MGIT medium containing starch (in red). The growth has been monitored with the BACTEC system. The control medium is indicated in black. (TIF) [file pntd.0002502.s001.tif]

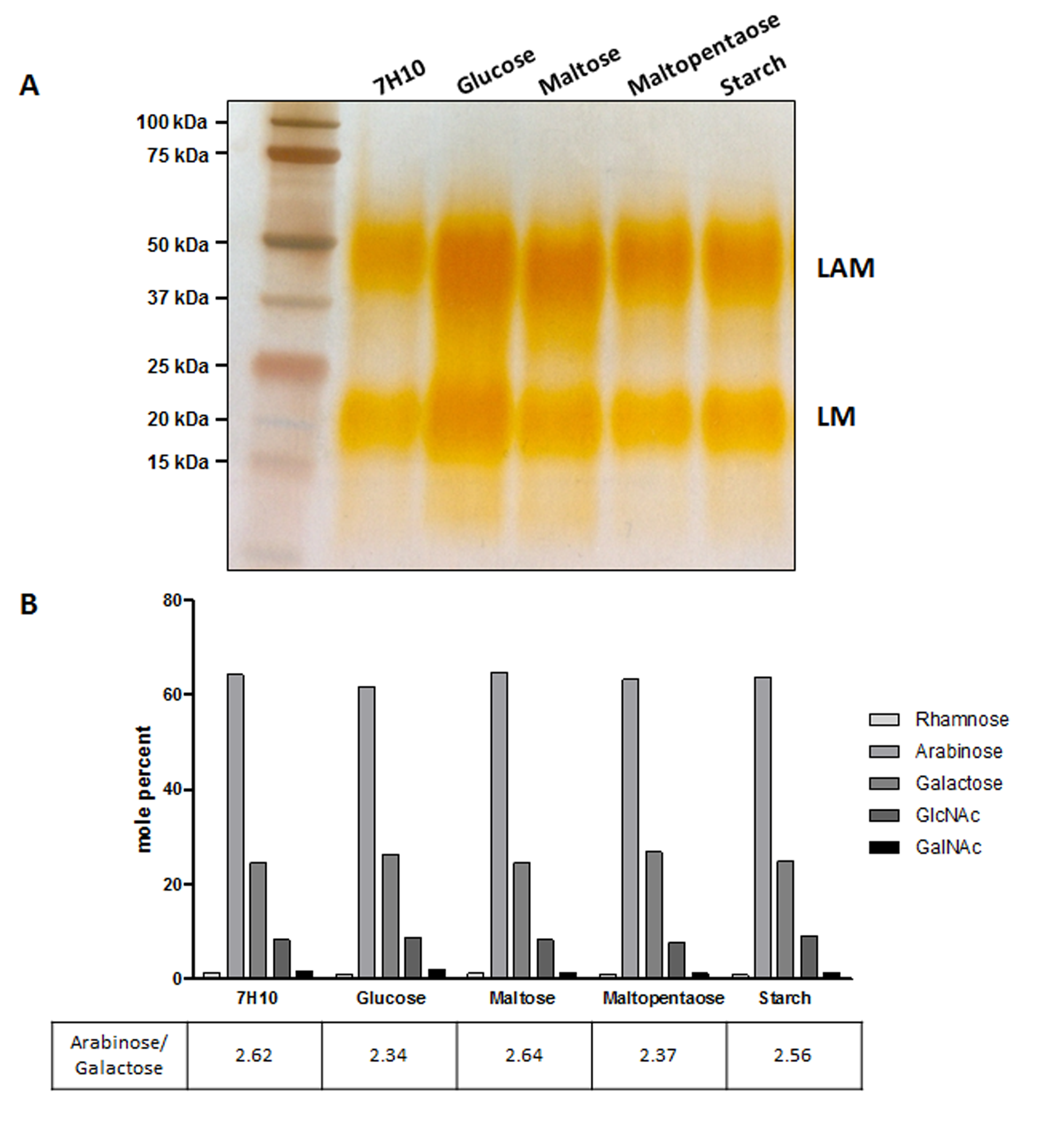

Supplement: Figure S2 — Analysis of lipomannan (LM), lipoarabinomannan (LAM) and mycolyl-arabinogalactan-peptidoglycan (mAGP). A- Analysis of LM and LAM from M. ulcerans 1615 strain grown on 7.5% carbohydrates-enriched 7H10. Lipoglycans were separated on a 10–20% Tricine gel and visualized by PAS staining. B- Monosaccharide composition of the mAGP complex of M. ulcerans 1615 strain grown under various conditions. mAGP was subjected to alditol acetate preparation. The mole percentage of each monosaccharide is indicated on the graph and the ratios of arabinose to galactose reflecting the composition of the major cell wall heteropolysaccharide, arabinogalactan, presented in the table. GlcNAc, N-acetyl-D-glucosamine; GalNAc, N-acetylgalactosamine. (TIF) [file pntd.0002502.s002.tif]

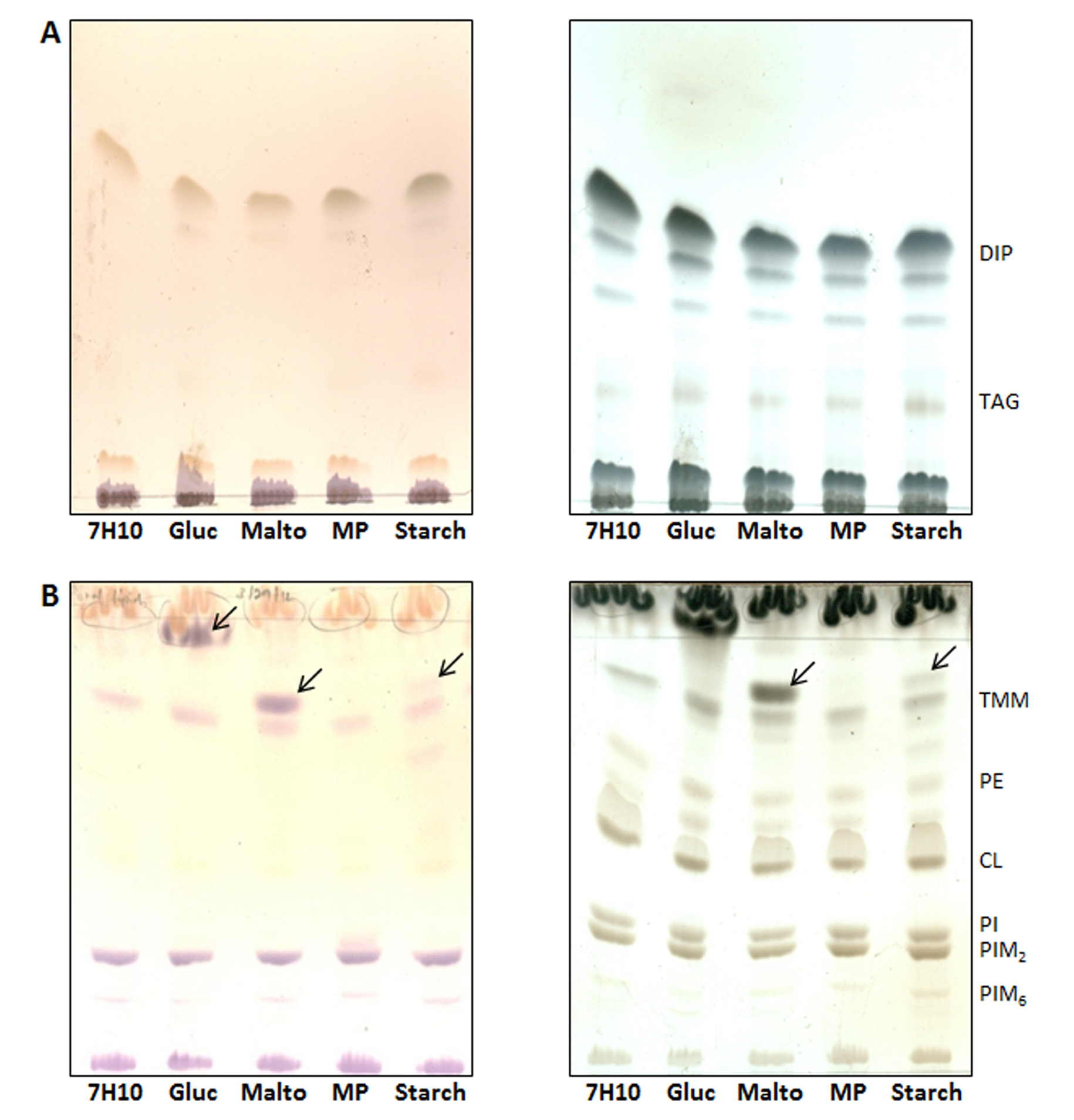

Supplement: Figure S3 — Total lipid analysis of M. ulcerans . Lipids from M. ulcerans were separated by TLC in two different solvent systems. A- Petroleum ether: ethyl acetate (98∶2; three developments). B- CHCl3/CH3OH/H2O (65∶25∶4). Lipids were revealed with alpha-naphthol (left panel) or cupric sulfate (right panel) followed by heating. Arrows highlight new lipid forms in 7.5% glucose, maltose and starch-enriched media compared to regular 7H10 medium. Gluc, 7H10 glucose; Malto, 7H10 maltose; MP, 7H10 maltopentaose; starch, 7H10 starch. CL, cardiolipin; DIP, phthiodiolone diphthioceranates and phenolphthiodiolone diphthioceranates; PE, phosphatidylethanolamine; PI, phosphatidylinositol; PIM, phosphatidylinositol mannosides; TAG, triacylglycerol; TMM, trehalose monomycolate. (TIF) [file pntd.0002502.s003.tif]

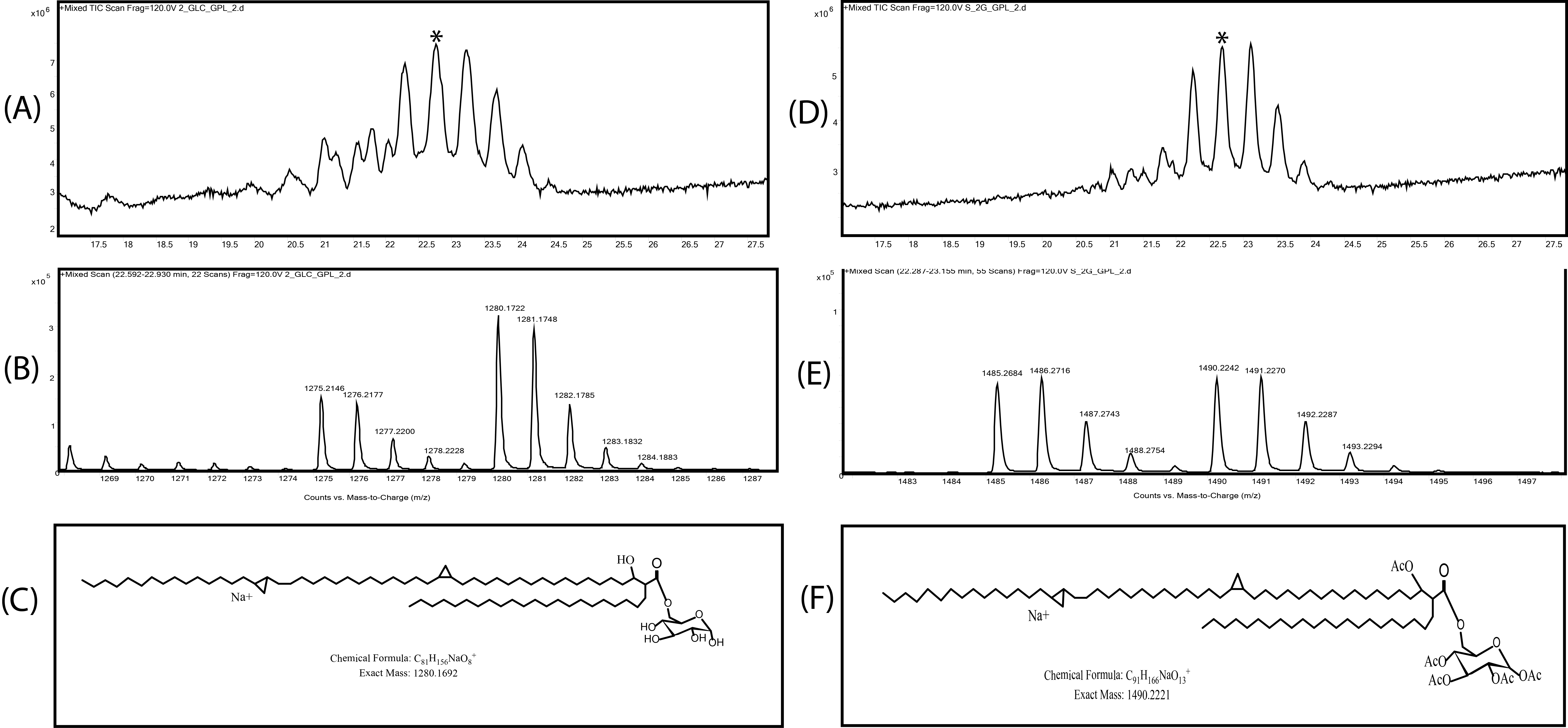

Supplement: Figure S4 — Identification of glucose monomycolate. (A) Total ion chromatogram of the LC/MS analysis of the TLC spot identified as GMM. (B) The mass spectrum of the major component of the starred peak in (A) showing a M+Na+ ion at m/z 1280.1722 (C81H156NaO8 with a calculated value of m/z of 1280.1670) and a M+NH4 + ion at m/z 1275.2146 (C81H156NaO8 with a calculated value of m/z of 1275.2139). (C) A structure consistent with the molecular weight data of (B) where the unsaturation is arbitrarily shown as cyclopropyl groups. (D) Total ion chromatogram of the LC/MS analysis of the TLC spot after per-O-acetylation. (E) The mass spectrum of the major component of the starred peak in (D) showing a M+Na+ ion at m/z 1490.2242 and a M+NH4 + ion at m/z 1485.2684. Both ions are consistent with the presence of five acetyl groups (one on the mycolyl hydroxyl group and four on C-1, C-2, C-3, and C-4 of the hexosyl residue thought to be glucose). (F) The structure as in (C) but with the 5 acetyl groups indicated. (JPG) [file pntd.0002502.s004.jpg]

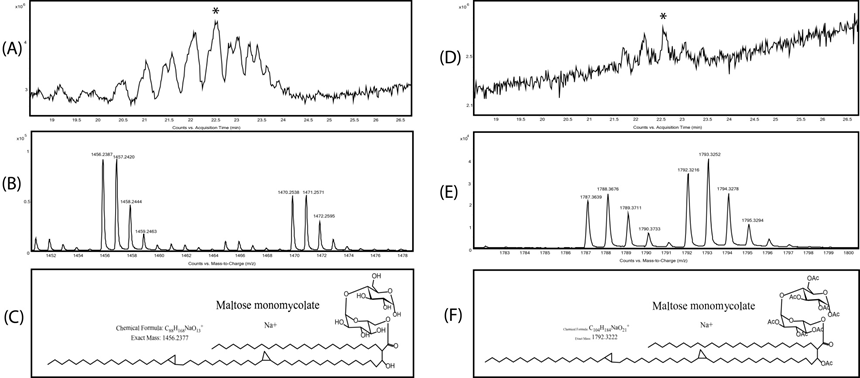

Supplement: Figure S5 — Identification of maltose monomycolate. (A) Total ion chromatogram of the LC/MS analysis of the TLC spot identified as maltose monomycolate. (B) The mass spectrum of the major component of the starred peak in (A) showing a M+Na+ ion at m/z 1456.2387 (C88H168NaO13 with a calculated value of m/z of 1456.2377) and also M+Na+ ion at m/z 1470.2538 (C89H168NaO13 with a calculated value of m/z of 1470.2534). (C) A structure consistent with the molecular weight data of (B) where the unsaturation is arbitrarily shown as cyclopropyl groups. (D) Total ion chromatogram of the LC/MS analysis of the TLC spot after per-O-acetylation. (E) The mass spectrum of the major component of the starred peak in (D) showing a M+Na+ ion at m/z 1792.3216 and a M+NH4 + ion at m/z 1787.3639. Both ions are consistent with the presence of 8 acetyl groups (one on the mycolyl hydroxyl group, three on C-2, C-3, and C-4 of the maltose linked to lipid, whereas the other four on C-4 linked maltose residue at C-1, C-2, C-3 and C-6 positions). (F) The predicted structure as in (C) but with the eight acetyl groups indicated. The identity of the di-hexosyl residue was not directly determined but assumed to be maltose based on the TLC migration properties of the glycolipid above TMM. (TIF) [file pntd.0002502.s005.tif]

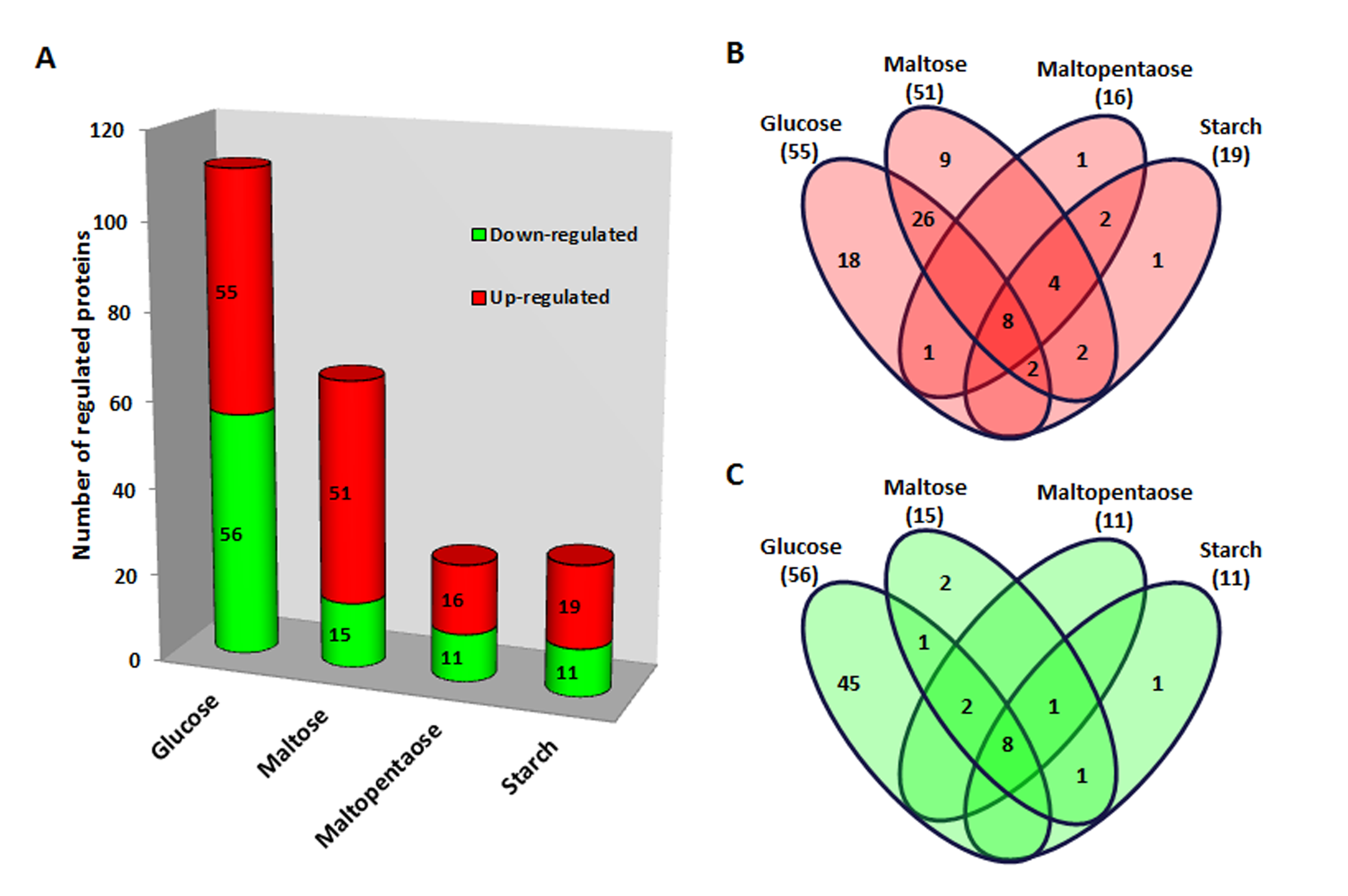

Supplement: Figure S6 — Repartition of M. ulcerans regulated proteins grown on 7.5% carbohydrates-enriched 7H10. A- Number of up- or down-regulated proteins in M. ulcerans grown on various media. Venn diagram showing the distribution of shared overproduced (B) or repressed (C) proteins among M. ulcerans grown on different media. (TIF) [file pntd.0002502.s006.tif]

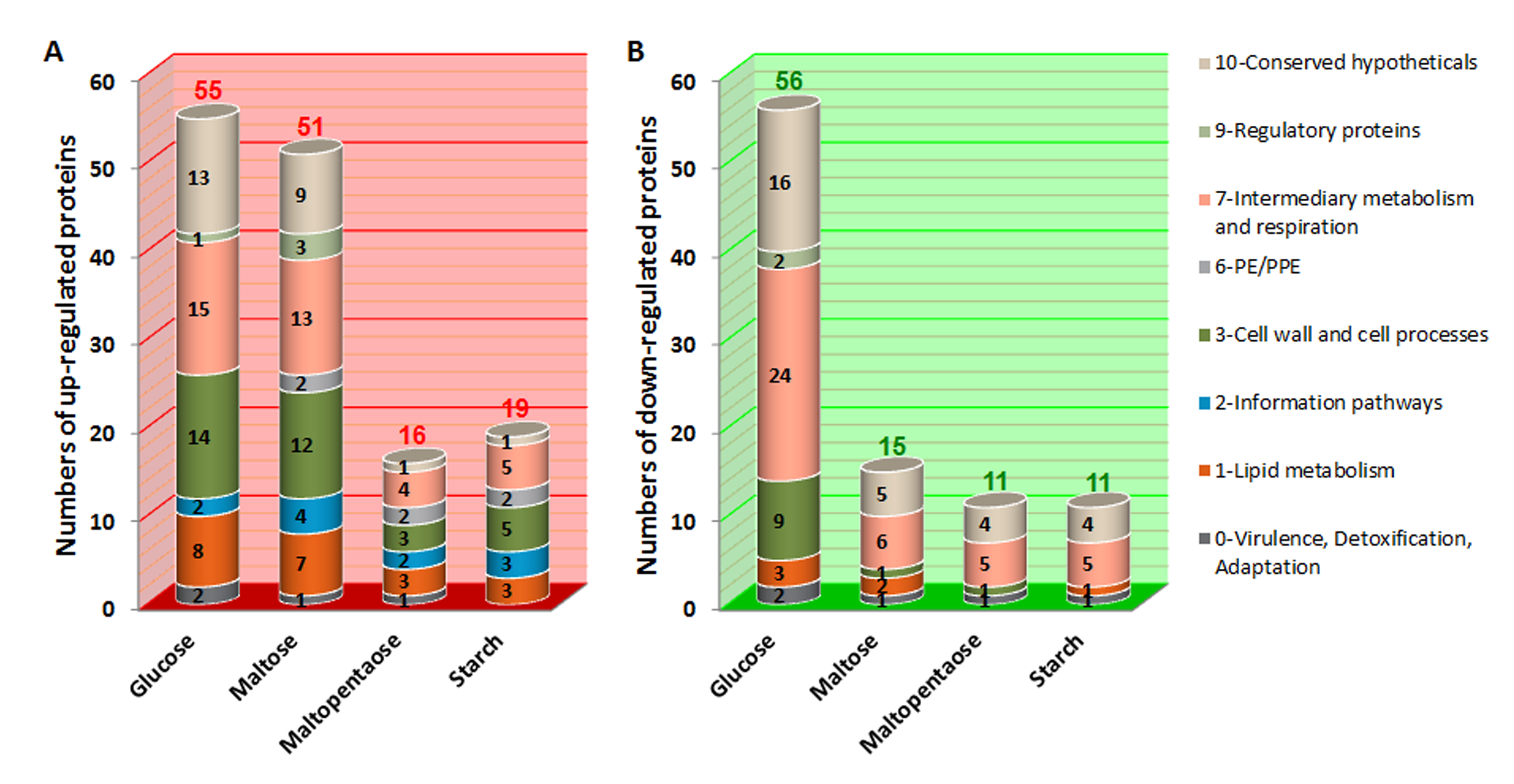

Supplement: Figure S7 — Distribution of functional protein categories of M. ulcerans regulated proteins grown on 7.5% carbohydrates-enriched 7H10. A- Distribution of functional categories of overproduced proteins. B- Distribution of functional categories of repressed proteins. The functional category of each protein was determined though BuruList website. They correspond to virulence, detoxification, adaptation; lipid metabolism; information pathways; cell wall and cell processes; PE/PPE; intermediary metabolism and respiration; regulatory proteins; conserved hypotheticals. (TIF) [file pntd.0002502.s007.tif]

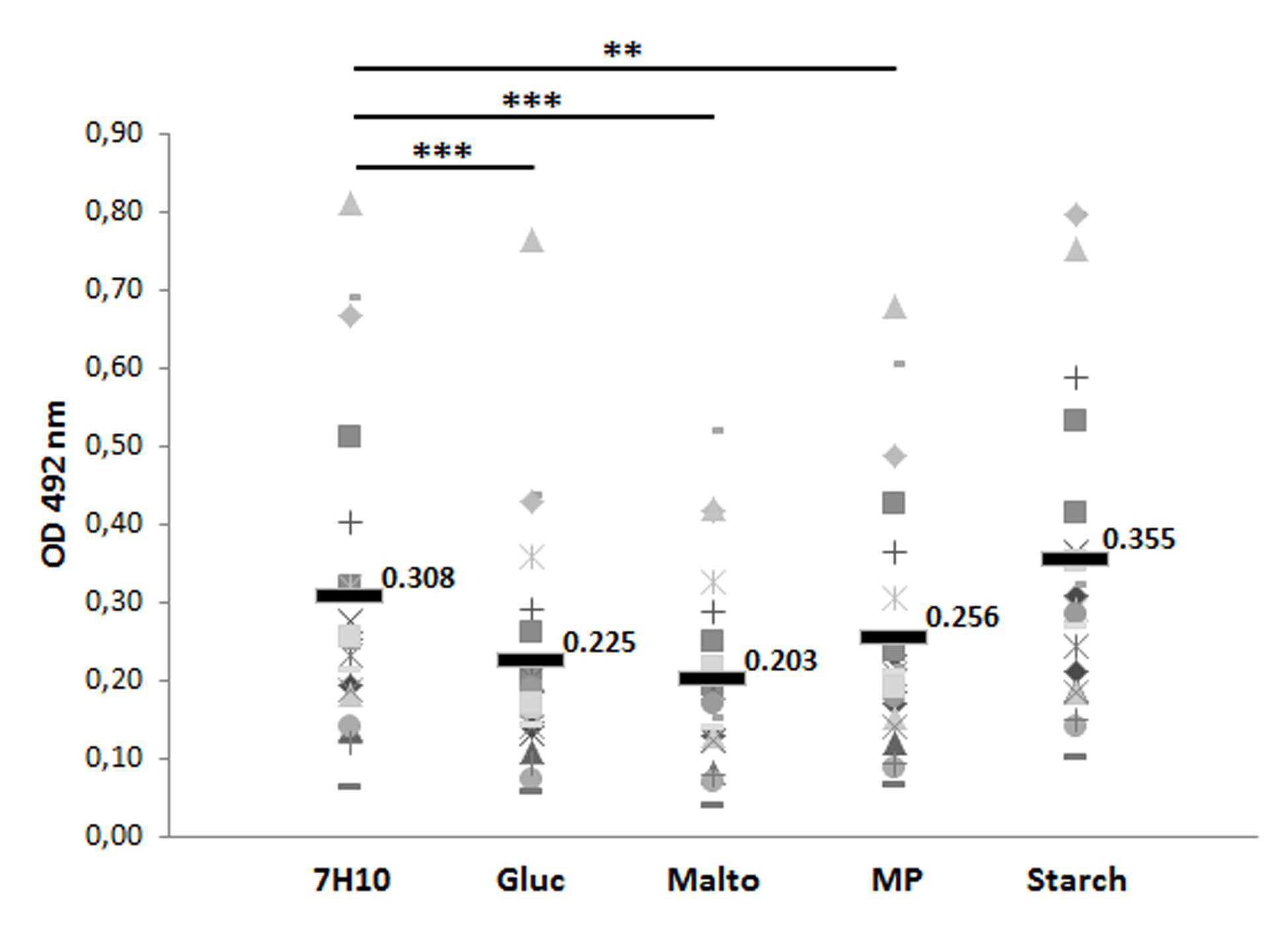

Supplement: Figure S8 — M. ulcerans antigens production in various conditions. Human IgG Binding to whole proteins lysate by ELISA Assay. Mean values are indicated by black thick drawbar. Each of the 21 tested sera has its own symbol. *** p<0.001. ** p<0.05. (TIF) [file pntd.0002502.s008.tif]

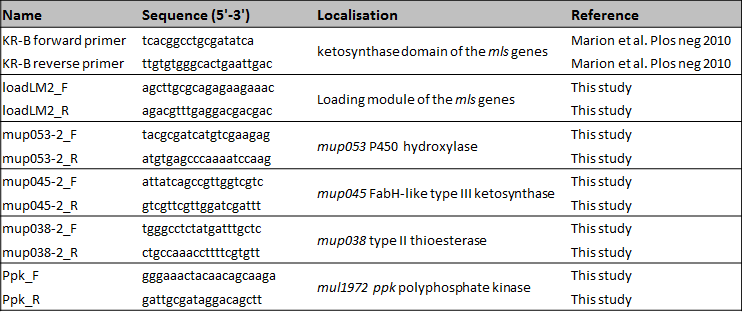

Supplement: Table S1 — Primers used in this study. (TIF) [file pntd.0002502.s010.tif]
