# Supplementary material for: Regulation of Mycolactone, the Mycobacterium ulcerans Toxin, Depends on Nutrient Source
Source: PLoS Negl Trop Dis. 2013 Nov 14;7(11):e2502. doi: 10.1371/journal.pntd.0002502 (PMC3828164; doi:10.1371/journal.pntd.0002502)
Supplement: Material and Methods S1 — Ethical aspect, alditol acetates of Arabinogalactan (AG), lipoarabinomannan (LAM) and Total cells, proteomic analysis by NanoLC MS/MS, human sera and ELISA analysis and supplementary references. (DOC) [file pntd.0002502.s009.doc]

**Supplementary Materials and Methods**

**Ethical aspect**

The Buruli Patients group consisted of 21 patients recruited from the Centre de Diagnostic et de Traitement de l'Ulcère de Buruli in Pobè, Benin. Fourteen out of 21 patients presented early clinical signs without ulceration (seven plaques, seven edemas) and seven patients with ulceration. The participants who had given their written consent were enrolled as volunteers during a study performed in 2006 , the protocols of which were approved by ethics Committee of Angers University Hospital (IRB) and by the Ministry of Health in Benin. Anonymized serum was prepared from 8 ml of blood from each participant and tested for potential HCV and HIV exposure using Access HIV-1/2 automated immunoassay (MDA/98/58) and Sanofi Diagnostics Pasteur Access anti-HCV automated immunoassay (plus update on five other anti-HCV assays—MDA/96/26). Diagnosis of M. ulcerans infection was made by Ziehl–Neelsen staining of material taken from swabs of the lesions or directly from the biopsy for the early form and confirmed by PCR .

**Alditol acetates of Arabinogalactan (AG), Lipoarabinomannan (LAM) and Total cells.** Total cells (lyophilized), mAGP and LAM were subjected to alditol acetates preparation as reported in the literature . Briefly, all the samples were acid hydrolyzed in 2 M TFA (250-500 µL) at 120 °C for 2 hours. Then, 10 µg of 3-*O-Me*-Glc (internal standard) was added and samples were completely dried under N_2_. The hydrolyzed sugars were subjected to overnight reduction using NaBD_4_ (500 μL) prepared at a concentration of 10 mg/mL in 1 M NH_4_OH in ethanol. After overnight reduction 20-50 μL of glacial acetic acid was added to remove excess NaBD_4_ and then 200 μL of acetic acid was added and completely evaporated to dryness. Approximately 500 μL of acetic anhydride was added to *per-O-*acetylate the reduced sugars at 120 °C for 2 hours. The samples were cooled, dried under N_2_ and then suspended in 3 mL of CHCl_3_/H_2_O. The chloroform layer was extracted, dried and further subjected to GC/MS analysis using Varian CP-3800 gas chromatograph equipped with a MS-320 mass spectrometer.

**Proteomic analysis by NanoLC MS/MS.** Triplicate protein samples (400 mg) were reduced in 5 mM THP for 30 min, and cysteines were alkylated in 10 mM iodoacetamide at RT in the dark for 30 min. Samples were diluted to 2 M urea with HEPES buffer pH 8.0, and trypsin (modified, sequencing grade, Roche) was added to the sample at a ratio of 1:50 enzyme/protein and allowed to digest overnight at 37 °C. Reaction was stopped by addition of 2% formic acid and peptides samples were cleaned up using a SupelCleanC18 cartridge (Sigma-Aldrich, St Louis, MO, USA), dried under low pressure in a SpeedVac and stored at -20 °C.

NanoLC MS/MS analysis were performed using an on-line system consisting of a micro-pump Agilent 1200 binary HPLC system (Agilent Technologies, Palo Alto, CA) coupled to an LTQ-Orbitrap XL hybrid mass spectrometer (ThermoFisher, San Jose, CA). Peptides were resuspended in mobile phase A (2.5 % acetonitrile, 0.1 % formic acid) and 8 μL was injected into a trapping column via an autosampler, at a flow rate of 5 μl/min. After loading, peptides were eluated onto a 75 μm (i.d.) × 10 cm capillary Picotip column (New Objective, Cambridge, MA), in-house-packed with C18 reverse-phase resin (Pursuit C18; 5 μm particles; 200-Å pore size; Varian, UK), and the flow rate was reduced to ca 100-200 nl/min using a vented column arrangement. Peptides were eluted into the MS using 140 min gradient from 0 % to 35 % mobile phase B (90 % acetonitrile, 10 % H_2_O, 0.025 % trifluoroacetic acid, 0.1 % formic acid). The mass spectrometer was operated in data-dependent mode, with a single MS scan (400-2000 m/z) followed by MS/MS scans in the linear ion trap on the 5 most abundant ions and dynamic excluded for 120 seconds. MS spectra data acquired for each samples were loaded to Progenesis LC-MS software (version 3.0, Nonlinear Dynamics, UK) for label free quantification and analysis. Profile data of the MS scans were transformed to peak lists with respective peak m/z values, intensities, abundances and m/z width. MS/MS spectra were treated similarly. For retention time alignment of the samples, the most complex sample was selected as a reference, and the retention times of the other samples were aligned automatically to a maximal overlay of all features. Features with only one charge or more than four charges were excluded from further analyses. Raw abundances of the remaining features were normalized to allow correction for factors resulting from experimental variation. Rank 1-5 MS/MS spectra were exported as Mascot generic file and used for peptide identification with MASCOT Version 2.3 (Matrix Science Ltd, UK) in the *M. ulcerans* Agy99 protein database (4241 sequences, released on February 2011). Search parameters were peptide mass tolerance of 7 ppm, and MS/MS tolerance of 0.4 Da allowing 1 missed cleavage. Carbamidomethylation of cysteine was set as a fixed modification, and oxidation of methionine, STY phosphorylation and N-protein acetylation were allowed as variable modification. Peptide assignments with an ion score cut-off of 20 and a significance threshold of *p*<0.05 were re-imported to Progenesis. After summing up the abundances of all peptides assigned to each protein, the results were grouped according to culture condition. We selected only proteins with 2-fold change to identify regulated proteins.

**Human sera and ELISA analysis.**

Total cell lysates (500 ng) from *M. ulcerans* were diluted in 100 μl 50 mM carbonate-bicarbonate buffer pH=9.6 (Sigma) and coated onto 96-well Nunc Maxisorp (ThermoScientific) plates by incubation overnight at 4 °C. The coated plates were then washed with PBST and saturated with 5 % skimmed milk PBS at RT for 3 h. After three washes in PBST, the samples were incubated for 1 h at RT with human sera diluted 1:100 in 1 % skimmed milk PBST. After three further washes, plate-bound human IgGs were detected using peroxidase-conjugated rabbit anti-human IgG (gamma chain specific) antibodies (Sigma) diluted 1:5,000 in 1 % skimmed milk PBST (1 h, RT) and OPD (Sigma). The diluted sera were tested in duplicate and the average absorbance at 492 nm was expressed in optical density units. One-way ANOVA analysis of variance was used to compare mean values between groups followed by Newman-Keuls multiple comparison test to detect significant mean differences between pairs of groups.

**Supplementary References**

1. Marsollier L, Brodin P, Jackson M, Kordulakova J, Tafelmeyer P, et al. (2007) Impact of *Mycobacterium ulcerans* biofilm on transmissibility to ecological niches and Buruli ulcer pathogenesis. PLoS Pathog 3: e62.

2. Marsollier L, Deniaux E, Brodin P, Marot A, Wondje CM, et al. (2007) Protection against *Mycobacterium ulcerans* lesion development by exposure to aquatic insect saliva. PLoS Med 4: e64.

3. Ross BC, Marino L, Oppedisano F, Edwards R, Robins-Browne RM, et al. (1997) Development of a PCR assay for rapid diagnosis of Mycobacterium ulcerans infection. J Clin Microbiol 35: 1696-1700.

4. Cassisa V, Chauty A, Marion E, Ardant MF, Eyangoh S, et al. (2010) Use of fine-needle aspiration for diagnosis of *Mycobacterium ulcerans* infection. J Clin Microbiol 48: 2263-2264.

5. Larrouy-Maumus G, Skovierova H, Dhouib R, Angala SK, Zuberogoitia S, et al. (2012) A small multidrug resistance-like transporter involved in the arabinosylation of arabinogalactan and lipoarabinomannan in mycobacteria. J Biol Chem 287: 39933-39941.
